# Tyrosinase Recovered from White Button Mushroom Waste: Extraction, Characterization, and Application in Casein Cross-Linking

**DOI:** 10.1021/acs.jafc.5c16655

**Published:** 2026-03-10

**Authors:** Trang Thuy Tran, Zhe Xu, John Coupland, Yi Zhang

**Affiliations:** Department of Food Science, 8082 The Pennsylvania State University, University Park, Pennsylvania 16802, United States

**Keywords:** tyrosinase, protein cross-linking, white button mushroom, mushroom waste, casein

## Abstract

Tyrosinase catalyzes the oxidation of mono- and diphenols to *o*-quinones, which can polymerize and covalently cross-link proteins, but the limited availability and high cost of purified tyrosinase limit broader use. This study recovered tyrosinase from white button mushroom (*Agaricus Bisporus*) stumps, an underutilized byproduct, and evaluated it for food protein modification. Proteomics identified multiple tyrosinase isozymes (AbPPO3, AbPPO4, AbPPO5), and the crude tyrosinase exhibited optimal activity at pH 7.5 and 45 °C, with a prominent 43 kDa protein band. Ammonium sulfate fractionation (50–70% saturation) increased specific activity; the 50% fraction achieved 4.4-fold purification with 47% activity recovery. Effects of chemical modulators, metal ions, salts, reductants, chelators, and inhibitors were systematically assessed. Endogenous proteolysis hindered cross-linking, but partial purification and EDTA/PMSF suppressed protease activity, enabling tyrosinase-catalyzed casein polymerization. These results demonstrate a cost-effective source to valorize mushroom waste into a tyrosinase biocatalyst for protein cross-linking.

## Graphical abstract

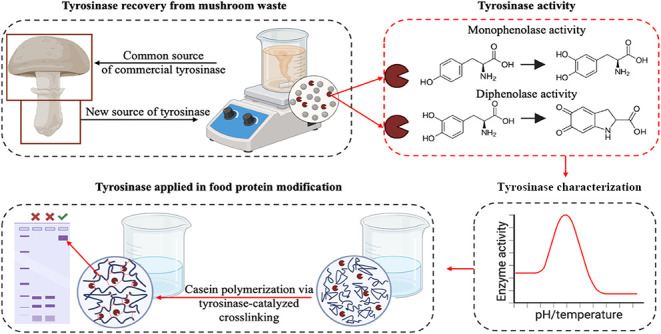

## 1 Introduction

White button mushrooms (*Agaricus bisporus*) are among the most widely consumed edible fungi globally and are highly valued for their nutritional and functional properties. In the US alone, the production of white button mushrooms reached 291,000 t in 2024, with Pennsylvania accounting for approximately 69% of the total production.^1^ While the caps and stems of white button mushrooms are used as food products, the stumps, the portion below the surface of the growth media, are typically considered agricultural waste. It is estimated that these stumps account for approximately 29% of the total mushroom biomass, corresponding to about 93,264 t annually.^2^ Some mushroom stumps are used as chicken feed,^2^ but the majority are discarded as landfill or through composting, which contributes to pollution problems and generates unpleasant odors.^3^ Recent research has highlighted the potential of converting agricultural byproducts into value-added biocatalysts, such as cellulases, xylanases, and peroxidases, providing both environmental and economic benefits.^4,5^ However, mushroom stumps remain largely unexplored despite being an abundant biological resource. Investigating their potential as a source of tyrosinase could offer a sustainable approach to enzyme production, while simultaneously reducing agricultural waste.

Tyrosinase (EC 1.14.18.1) is a copper-containing oxidase widely found in mushrooms, with the white button mushroom being the primary source for commercial extracts. Tyrosinase consists of two heavy chains (43–48 kDa) containing the active site, and two light chains (13–14 kDa), which must be removed by proteolytic cleavage to activate the enzyme.^6^ At least six tyrosinase isozymes have been identified in mushroom tissues (skin and flesh of the cap, gills, velum, and stump), which differ in molecular weight, pH optima, substrate specificity, inhibitor sensitivity, and catalytic properties.^7–9^ While commercial tyrosinase is typically extracted from mushroom fruiting bodies, the potential of mushroom stumps as a source of tyrosinase isozymes has not been previously explored. We hypothesize that tyrosinases derived from stumps may exhibit distinct enzymatic properties due to their development in a unique microenvironment beneath the growing medium. Given the high cost and limited availability of commercial tyrosinase, the valorization of this underutilized biomass offers a promising, sustainable, and cost-effective alternative source for tyrosinases.

Tyrosinase catalyzes both monophenolase and diphenolase reactions, oxidizing phenolic compounds to highly reactive quinones. These quinones subsequently undergo a series of nonenzymatic reactions to produce melanin, which contributes to the unwanted browning in fresh fruits and vegetables. The quinones can also covalently cross-link proteins by reacting with nucleophilic amino acid side chains such as those of lysine (amine) and cysteine (sulfhydryl) (Figure S1). Tyrosinase-catalyzed cross-linking of proteins has been used to modify the properties of food colloids, enhance the stability of protein-based emulsions, and create biobased food packaging.^10–12^

Casein, the primary protein in milk, is widely used as a food ingredient. Recent studies have explored its potential in cross-linked networks of casein for biopackaging, gel formation, and emulsion stabilization. Various cross-linking methods have been investigated, including enzymatic approaches using tyrosinase, transglutaminase, and peroxidase, as well as nonenzymatic methods including pH or heat treatments.^13,14^ For example, tyrosinase has been used to cross-link sodium caseinate to form protein nanoparticles,^15^ to create biofilms through caseinate-polyphenol-chitosan interactions,^16^ and to generate protein gels from blends of whey protein isolates, calcium caseinate, and alginic acid.^17^ While purified mushroom cap and stem tyrosinase have been widely used in these studies, crude tyrosinase extracted from mushroom stumps has not been explored for casein cross-linking.

This study aims to (1) extract and characterize tyrosinase from white button mushroom stumps and (2) demonstrate its ability to catalyze food protein cross-linking. This is the first study to explore the potential of tyrosinase from mushroom processing waste as a sustainable approach for value-added food applications.

## 2 Materials and Methods

### 2.1 Materials and Chemicals

White button mushrooms were grown at the Mushroom Research Center at the Pennsylvania State University (University Park, PA, USA) in soil pH of 7.2. All chemicals used, including 3,4-dihydroxy-l-phenylalanine (l-DOPA), phenylmethanesulfonyl fluoride (PMSF), ethylenedinitrilotetraacetic acid (EDTA), β-mercaptoethanol (β-ME), and DL-dithiothreitol (DTT), were purchased from Sigma-Aldrich Inc. (St. Louis, MO, USA). Commercial tyrosinase was purchased from Sigma-Aldrich (T3824, CAS 9002–10–2) as a reference, which is derived from mushrooms.

### 2.2 Crude Tyrosinase Extraction

White button mushroom stumps were collected on the harvest day, cleaned by manually removing the mycelium and solid culturing medium, and then rinsed under tap water. The samples were dried using paper towels, then vacuum-sealed in clear plastic bags, and stored at −20 °C until required for use. To extract crude tyrosinase, the stumps were blended for 2 min with sodium phosphate buffer (50 mM, pH 6.5) in a 3:5 (w/v) ratio. The homogenate was transferred into a prechilled beaker, stirred at 4 °C for 1 h, and then centrifuged for 20 min at 11,000*g*, 4 °C. The supernatant was used as “crude tyrosinase” and was stored at 4 °C for up to a week.

### 2.3 Ammonium Sulfate Precipitation (ASP) Fractionation

All steps were carried out at 4 °C. The crude tyrosinase was mixed with solid anhydrous ammonium sulfate to reach the desired saturation (30, 40, 50, 60, 70, or 80%), stirred at 4 °C for 1 h, and then centrifuged for 20 min at 11,000*g*, 4 °C to obtain the partial purified tyrosinase pellet. The pellets from each fraction were resuspended in sodium phosphate buffer (50 mM, pH 6.5) and used in subsequent applications.

### 2.4 Protein Quantification and Enzyme Activity Assay

Protein concentration was determined using the bicinchoninic acid (BCA) assay^18^ with bovine serum albumin (BSA) as a standard.

Tyrosinase activity was determined using a minor modification of the methods of Sigma-Aldrich^19^ for monophenolase activity, and Pretzler and co-workers^20^ for diphenolase activity. (1) For monophenolase activity, a 2 mL reaction mixture containing 1990 μL of 1 mM l-tyrosine prepared in 50 mM sodium phosphate buffer (pH 6.5) and 10 μL of enzyme sample was incubated at 25 °C for 10 min to allow for the lag phase before measuring the rate of absorbance change. One unit of monophenolase activity is defined as an amount of enzyme that produces a Δ*A*_280_ of 0.001 per min in the above condition. (2) For diphenolase activity, a 2 mL mixture consisting of 1990 μL of 1 mM l-DOPA in 50 mM sodium phosphate buffer (pH 6.5) and 10 μL of enzyme sample was prepared, and the change in absorbance was measured immediately after mixing at 25 °C. The rate of the enzymatic reaction was determined as the increase in absorbance of l-DOPA (280 nm) or dopachrome (475 nm) over 3 min. One unit of tyrosinase activity (diphenolase activity) is defined as the amount of enzyme required to catalyze the formation of 1 μmol of dopachrome per min from l-DOPA.

Protease activity was measured using Sigma’s nonspecific protease activity assay^21^ with the minor modification of using Whatman No.1 filter paper instead of a 0.45 μM polyether sulfone syringe filter. One protease unit is defined as the amount (in μmol) of tyrosine equivalents released from casein per minute.

### 2.5 Optimal pH and pH Stability

To determine the optimal pH for tyrosinase activity, l-DOPA was prepared in buffers with pH values ranging from 3.0 to 10.0. The following buffers were used: citrate buffer (pH 3.0, 6.2), phosphate buffer (pH 5.8, 8.0), and carbonate-bicarbonate buffer (pH 9.2–10.6). The tyrosinase activity assay and specific activity were then measured as described above (Section 2.4).

The pH stability of tyrosinase was determined using the method of Han et al.^22^ with minor modifications. The crude tyrosinase was concentrated 10 times by ultrafiltration with a 10 kDa cutoff membrane to eliminate the phosphate buffer solution. The concentrated crude tyrosinase (0.1 mL) was incubated at 4 °C for 24 h with a buffer solution (9.9 mL) at various pHs as described previously. The tyrosinase activity assay was conducted at pH 6.5, as described above (Section 2.4). The residual activity (%) was calculated using the tyrosinase initial activity under each pH condition at 0 h as 100%.

### 2.6 Optimal Temperature and Thermal Stability

Tyrosinase activity was measured as a function of temperature (0–70 °C) using the methods described previously (Section 2.4). Tyrosinase thermal stability was assessed using the method of Kolcuoğlu^23^ with minor modifications. Briefly, crude tyrosinase (100 μL) was incubated at 0–70 °C for 0–24 h and then rapidly cooled in tap water to 25 °C. The residual activity (%) was calculated using the tyrosinase initial activity at 21 °C, pH 6.5, and 0 h as 100%.

### 2.7 Enzyme Kinetics

Tyrosinase activity was measured as a function of l-DOPA concentration (0–10 mM). The data obtained were plotted using the Lineweaver–Burk plot, and the Michaelis–Menten equation was used to determine *K*_m_ (the Michaelis constant) and *V*_max_ (the maximum reaction rate).

### 2.8 Effects of Chemical Reagents and Metal Ions on Enzyme Activity

The effect of selected metal ions (CuSO_4_, FeSO_4_, MnSO_4_, ZnSO_4_, NaCl)^24^ and chemical reagents (β-mercaptoethanol, EDTA, DTT, SDS, PMSF, l-ascorbic acid, citric acid, and l-cysteine)^25^ on tyrosinase activity was measured. Among these, PMSF and EDTA are commonly used as protease inhibitors. Briefly, each compound was individually mixed with l-DOPA to obtain targeted concentrations and then added to the enzyme activity assay. Residual activity was calculated as the ratio of the measured activity to that of the original control.

### 2.9 Proteomic Analysis Using LC–MSMS

Protein bands of interest were cut from the SDS-PAGE gel and submitted for in-gel digestion and protein identification via LC–MSMS at the PSU Proteomics and Mass Spectrometry Core Facility (University Park, PA, USA). Gel bands were destained, reduced, alkylated, and subjected to trypsin digestion following the standard protocols used by the facility. Peptides were extracted and analyzed using nanoelectrospray ionization liquid chromatography with tandem mass spectrometry (Thermo Orbitrap Eclipse, Thermo Fisher Scientific). The data were analyzed with Proteome Discoverer v 2.5. Raw spectra were processed following the Core Facility’s established bioinformatics workflow.^26–28^ Protein identifications were assigned based on matching MS2 fragmentation patterns to theoretical peptide spectra, and results were reported with protein accession numbers, protein description, sequence coverage (%), number (No.) of peptides, number (No.) of unique peptides, number of peptide spectra mapping (No. PSM), number of amino acids (No. AAs), and MW (kDa).

### 2.10 Casein Cross-Linking Using Crude Tyrosinase

A casein solution was prepared by dissolving sodium caseinate in phosphate buffer (pH 7.0, 50 mM) and heating the mixture to 80 °C for 30 min with constant stirring. The solution was then cooled to room temperature prior to use.

To assess the effect of proteases in the crude tyrosinase extract on casein, a reaction mixture (2 mL) containing 1000 nU of crude tyrosinase and 0.2% (w/v) casein was incubated at 30 °C with shaking. Samples were collected every 20 min over a 2 h period, mixed with SDS-PAGE loading buffer containing β-ME, and then boiled for 5 min to terminate enzymatic reactions. A control group containing only 0.2% casein (no enzyme) was included.

To evaluate the effectiveness of ammonium sulfate precipitation in reducing protease contamination, partially purified tyrosinase fractions (50, 60, 70, 50–70, and 60–70% saturation) were tested. Each fraction (containing 1000 nU tyrosinase activity) was added to 0.2% casein and shake-incubated at 30 °C for 2 h. Reactions were stopped by mixing samples with SDS-PAGE loading buffer and boiling. Controls included 2 mL of 0.2% casein alone and 2 mL of heat-inactivated enzyme fractions combined with 0.2% casein.

To investigate the effect of protease inhibitors on casein degradation and cross-linking, crude tyrosinase extract was preincubated with PMSF (1 and 2 mM) or EDTA (5 and 10 mM) for 10 min at 4 °C. Following inhibitor treatment, 2% (w/v) casein was added to each sample and incubated at 30 °C overnight with shaking. Reactions were stopped by boiling them with SDS-PAGE loading buffer.

Controls for cross-linking experiments are described as follows. Three negative controls: 2 mL of 2% casein alone, 2 mL of inactive tyrosinase with 2% casein, and 2 mL of inactive tyrosinase (pretreated with 2 mM PMSF or 10 mM EDTA) with 2% casein. A positive control: 2 mL of 2000 nU of commercial tyrosinase with 2% casein. To prepare heat-inactive tyrosinase controls, crude or partially purified tyrosinase samples were boiled at 100 °C for 30 min and then cooled to room temperature before use.

### 2.11 Electrophoresis Study

SDS-PAGE was performed at room temperature on a BIO-RAD Mini-PROTEAN Tetra system (Bio-Rad, USA). Samples were run either on a 5% stacking gel and 12% resolving gel or a 4–15% precast gel (Bio-Rad, USA), followed by staining with Coomassie Blue R-250 to assess the protein profiles of each sample.

### 2.12 Statistical Analysis

All experiments were performed in triplicate. Statistical analyses were conducted using Minitab (version 20). Descriptive statistics, including mean and standard deviation, were calculated for each group. One-way ANOVA was used to assess significant differences among group means. If ANOVA indicated significance (*p* < 0.05), Tukey’s HSD test was performed for pairwise comparisons at a 95% confidence level.

## 3 Results and Discussion

### 3.1 Tyrosinase Extraction and Partial Purification

#### 3.1.1 Crude Tyrosinase

The protein profile in the crude mushroom stump extract was observed by SDS-PAGE gel electrophoresis and is shown in Figure 1a. Crude tyrosinase (Lane 2) contained high-MW bands (43 and 45 kDa), and low-molecular-weight bands (∼26 and 14 kDa) similar to those in the commercial tyrosinase (Lane 1) (44 and 46 kDa, as well as 26 and 14 kDa). Tyrosinase is present as a latent form with two heavy chains containing the active site (MW 43–48 kDa) and two light chains (MW of 13–14 kDa).^7,29^ Therefore, the 43 and 45 kDa in crude tyrosinase are likely to correspond to the heavy chains, and the 14 kDa to the light chains. The heavy chains in crude tyrosinase had a slightly lower MW than that of commercial tyrosinase. There are six tyrosinase isoenzymes (*Ab*PPO1-*Ab*PPO6) present in button mushrooms with a range of MW (43–47 kDa). It seems that the crude tyrosinase from mushroom stumps contained the AbPPO4 (43.2–44 kDa) and AbPPO1 or 2 (45 kDa) isozymes, while the commercial tyrosinase from mushroom caps had AbPPO4 (43.2–44 kDa) and AbPPO3 (45.3 kDa). The difference in composition between the crude stump extract and the commercial tyrosinase may be due to differences in isoenzyme expression in different mushroom tissues.^7,9,30^ Both the commercial and crude tyrosinases also contained an unidentified protein band ∼24 kDa. Crude tyrosinase (Lane 2) also contained several other unidentified proteins. Nonetheless, the intensity of the tyrosinase-specific bands indicates that tyrosinase constitutes a major component of the crude extract.

**Figure 1:**
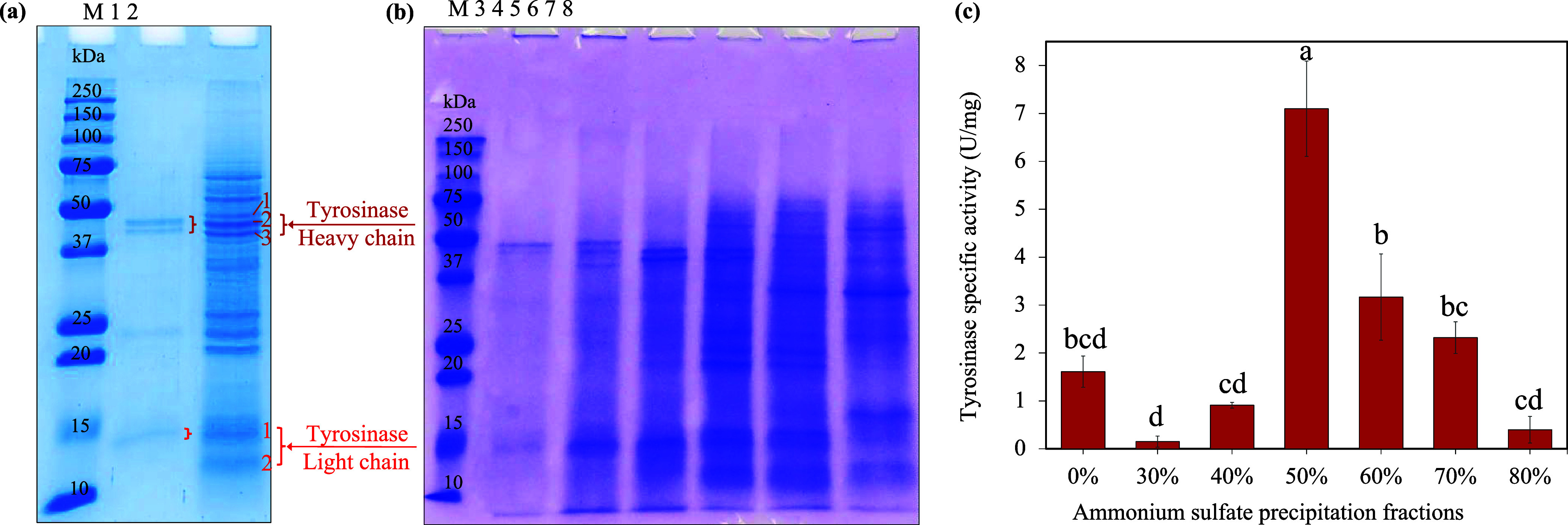
Protein profile and specific activity of tyrosinase from button mushroom stumps. (a) SDS-PAGE profile of tyrosinase recovered from mushroom stumps. Lane M: Protein marker, Lane 1: Commercial tyrosinase, Lane 2: Crude tyrosinase (heavy chains 1, 2, 3 and Light chains 1, 2). (b) SDS-PAGE profile of partially purified tyrosinase. Lane 3: 30% saturation fraction, Lane 4: 40% saturation fraction, Lane 5: 50% saturation fraction, Lane 6: 60% saturation fraction, Lane 7: 70% saturation fraction, Lane 8:80% saturation fraction; (c) Specific activity of crude tyrosinase and partially purified tyrosinase (30–80% saturation fractions). Samples with different superscript letters are significantly different (*p* < 0.05).

Monophenolase and diphenolase activities were quantified based on the rate of product formation from l-tyrosine and l-DOPA, respectively. As shown in Table 1, both crude and commercial tyrosinases exhibited measurable monophenolase and diphenolase activities. Monophenolase activity is considered a distinguishing feature of tyrosinase, as it measures the oxidation of monophenols like l-tyrosine amino acid. Therefore, enzymes with higher monophenolase activity are regarded as more promising candidates for protein cross-linking applications. However, the absolute value of the monophenolase activities from the two samples could not be meaningfully compared as their degree of purification differed. Instead, the ratio of monophenolase to diphenolase activity was used to compare the relative monophenolase activities. The crude tyrosinase had a higher ratio (3544 U monophenolase/U diphenolase) than the commercial tyrosinase (3083 U monophenolase/U diphenolase), suggesting the active enzyme present had a greater catalytic activity for monophenol compounds. This difference may be attributed to variations in isoenzyme composition. Pretzler et al. reported variations in substrate affinity among six *Agaricus bisporus* tyrosinase isoenzymes: *Ab*PPO2 and *Ab*PPO5 showed the highest affinity toward both l-tyrosine and l-DOPA, whereas *Ab*PPO3 displayed no catalytic activity with l-tyrosine.^7^ The higher monophenolase activity in crude tyrosinase suggests its greater efficiency in utilizing l-tyrosine, which could enhance its potential for protein cross-linking applications.

**Table 1:** Monophenolase and Diphenolase Activities In Crude Tyrosinase Versus Commercial Tyrosinase

	monophenolase activity (U)	diphenolase activity (U)	monophenolase/diphenolase activity
commercial tyrosinase	92.5	0.03	3083
crude tyrosinase	319	0.09	3544

The specific tyrosinase activity in crude tyrosinase extract was 1.61 U/mg (Table 2), which is lower than the previously reported values for similar crude tyrosinases from portabella mushroom caps (19 U/mg)^31^ and from the whole button mushrooms (3.189 U/mg).^32^ However, due to differences in methodology and the activity assay methods employed across studies, a direct comparison of specific activities is not reliable. Besides that, the crude extract also had a lower specific activity than the commercial tyrosinase studied here (4.02 U/mg). Thus, it may be necessary to perform further purification steps to remove impurities and improve the specific activity of the tyrosinase.

**Table 2:** Partial Purification of Tyrosinase Using Ammonium Sulfate Precipitation (ASP)

ASP fractions	fraction volume (mL)	total activity (U)	total protein (mg/mL)	specific activity (U/mg)	fold purification	percentage of yield (%)
Crude	50	238.75	147.53	1.61	1.00	100
30%	2	1.35	9.46	0.15	0.09	0.57
40%	2	4.70	5.19	0.91	0.56	1.97
50%	2	112.50	15.77	7.10	4.41	47.12
60%	2	36.40	11.39	3.17	1.97	15.25
70%	2	27.40	11.56	2.32	1.44	11.48
80%	2	3.60	9.46	0.40	0.25	1.51
50–70%	3	114.00	39.82	2.87	1.78	47.75
60–70%	3	68.25	28.68	2.41	1.50	28.59

#### 3.1.2 Partially Purified Tyrosinases

Crude tyrosinase was partially purified by ammonium sulfate precipitation (30, 40, 50, 60, 70, 80, 50–70, and 50–60% saturation). The specific activity, percentage of yield, and fold purification of each fraction are listed in Table 2. The 50% saturated fraction had the highest specific activity (7.1 U/mg), corresponding to a 4.41-fold purification and 47% enzyme recovery. The 60 and 70% saturated fractions also had high tyrosinase purification (1.97-fold and 1.44-fold purification, respectively) while a combination of the 50–70% ammonium sulfate fractions showed a specific activity of 1.78-fold and 48% enzyme recovery. The few previous studies on tyrosinase purification via ammonium sulfate precipitation used different saturation ranges (45–80, 0–60, and 30–60%) and achieved different fold purifications (3.47-fold, 1.91-fold, and 3.95-fold, respectively).^33–35^

The protein profiles of the partially purified tyrosinases were confirmed using SDS-PAGE (Figure 1b), and the corresponding specific activities were determined (Figure 1c). The saturated fractions of 50 and 60% showed the clearest and most intense band of tyrosinase (∼43 kDa), while the 50% saturation also showed the fewest impurity proteins, which aligned with its highest specific activity. Ammonium sulfate precipitation is a cost-effective initial step sometimes used as part of a more comprehensive, multistep enzyme purification process.^34,36,37^ The high price of commercial tyrosinase is likely due to the high cost of these additional purification steps. It is possible that the simple, low-cost process used achieved adequate purity to bring an affordable and functional tyrosinase product to the market.

### 3.2 Enzymological Properties of Tyrosinase

#### 3.2.1 Optimal pH and pH Stability

The effect of pH on the activity of crude tyrosinase is shown in Figure 2a. The enzyme had activity over the pH range 4.0–10.0, with a maximum at pH 7.5 and a broad optimal range of pH 6.0–8.0. In comparison, commercial tyrosinase had an optimal pH of 7.0–7.5 with a similar optimal pH range (6.0–8.0). The differences may be attributed to the presence of different tyrosinase isoenzymes.^7^ To assess the pH stability of the crude tyrosinase extract, samples were incubated at 4 °C for 24 h under different pH conditions and brought back to pH 6.5 to measure the enzyme activity. The tyrosinase retained its original activity when incubated at pH 7.0–10.0, while only a 10–20% loss of activity was observed at moderately acidic conditions (pH 5.0–6.5) (Figure 2b). At extreme acidic conditions (pH 3–4), the enzyme lost all activity within 2 h (Figure S2). The natural pH of mushroom stumps is neutral (∼7.2), which may explain the enzyme’s stability in the pH range of 7.0–7.5.

**Figure 2:**
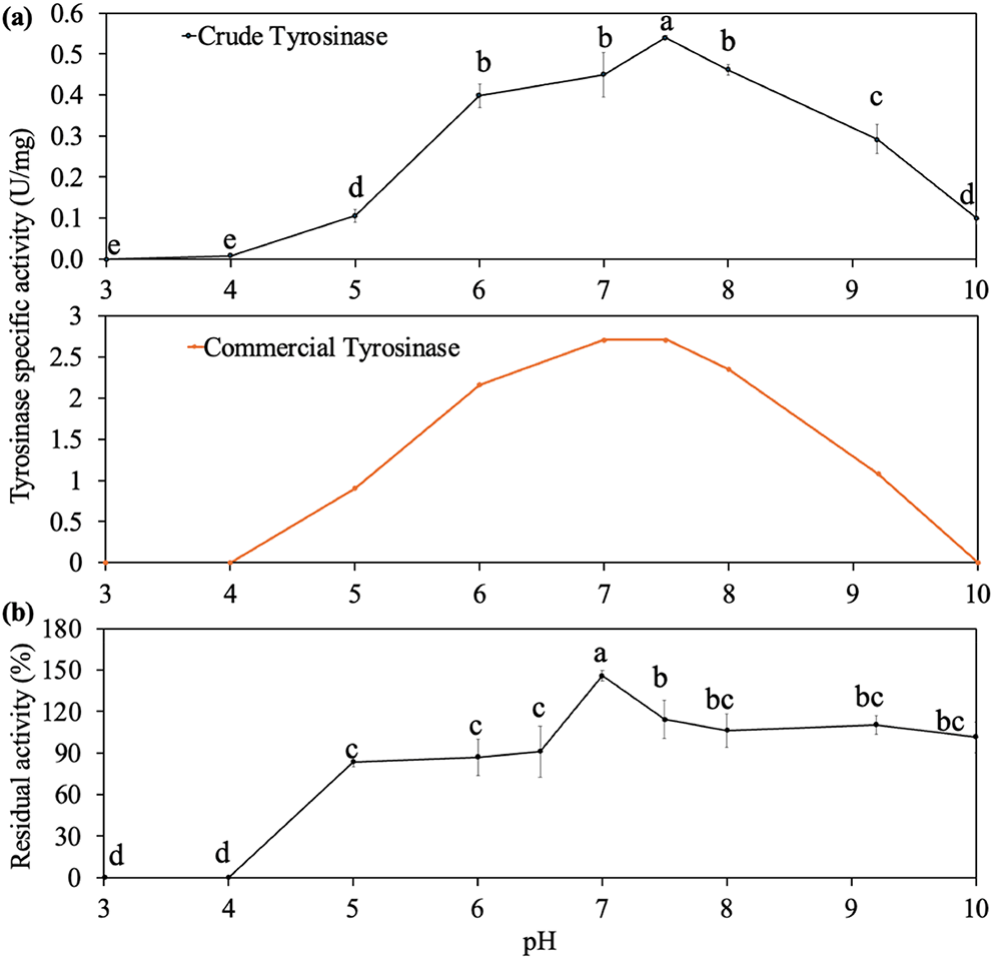
Effect of pH on (a) crude and commercial tyrosinase optimum activity; (b) pH stability of tyrosinase after 24 h. 100% of residual activity was taken as the original activity of each pH at 0 h. Samples with different superscript letters are significantly different (*p* < 0.05).

Mushroom tyrosinase has been shown to undergo dissociation and unfolding under acidic conditions (pH 2.0), leading to exposure of hydrophobic residues, destabilization of the active site, and reduced enzymatic activity.^38^ Similarly, alkaline conditions (pH 9.0–10.0) cause the transition through structural intermediates that lead to the reduction of enzyme activity.^39^ Devi et al. observed two reversible conformations of tyrosinase at acidic and neutral pH, each with different enzyme–substrate interactions.^39^ In particular, tyrosinase was inhibited by excess tyrosine at pH 6.8, an effect that was absent at pH 5.0 but which reappeared upon returning to pH 6.8, suggesting reversible, pH-induced structural changes. These results explained the tyrosinase behavior in the optimal pH and pH stability assay. Although only a few studies have examined the stability of *Agaricus bisporus* tyrosinase under different pH conditions, the stability of tyrosinase from other sources has been reported. In comparison to these reports in the literature, tyrosinase from white button mushroom stumps exhibits higher stability under alkaline conditions. For example, *Pleurotus ostreatus* fungi tyrosinase lost 65% activity after 24 h at pH 7.0, while *Lactarius piperatus* (L.) Pers. mushroom tyrosinase remained stable at pH 5.0, 6.0, and 7.0 over 24 h, with only a 10–20% activity loss at pH 3.0, 4.0, 8.0, and 9.0.^35,40^ Similarly, tyrosinase from a thermophilic bacterium lost 25% activity after 20 h in the pH range of 8.5–10.0.^41^ In comparison to these findings, the tyrosinase extracted from white button mushroom stumps in the present work appears to show a higher stability under alkaline conditions.

#### 3.2.2 Optimal Temperatures and Thermal Stability

Tyrosinase from white button mushroom stumps exhibited enzymatic activity across a broad temperature range (4 to 60 °C), with an optimal temperature of 45 °C (Figure 3a). Although the optimum temperature was close to that of the commercial tyrosinase (45 °C), the crude mushroom extract had a broader optimal range (30–50 °C vs 40–50 °C). The optimal temperatures of both the crude extract and the commercial tyrosinase were higher than values previously reported in the literature for white button mushroom tyrosinase (20 and 35 °C).^35,42,43^ The broader temperature adaptability of the crude extract may be due to differences in isozyme composition, similar to the broader pH adaptability described above. However, the temperature dependence of individual isozymes has not yet been studied.

**Figure 3:**
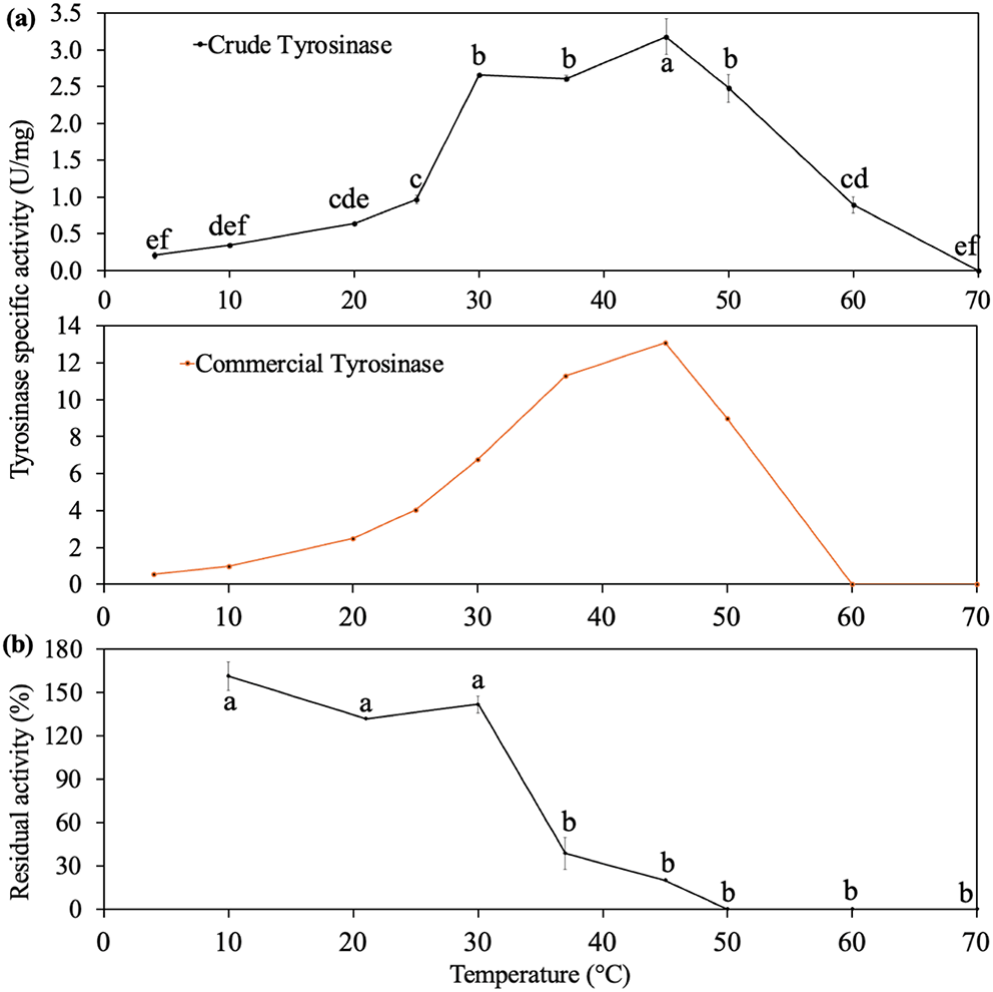
Effect of temperature on (a) crude and commercial tyrosinase activity; (b) thermal stability of tyrosinase after 24 h. The residual activity (%) was calculated using the tyrosinase initial activity at 21 °C, pH 6.5, and 0 h as 100%. Samples with different superscript letters are significantly different (*p* < 0.05).

The crude tyrosinase exhibited thermal stability between 4 and 30 °C over a 24 h period, with the highest stability observed at 10 and 30 °C (Figure 3b). However, at 37 and 45 °C, the enzyme was stable for the first 1.5 h, but lost 60–80% of its activity within 24 h (Figure S2). At higher temperatures (50–70 °C), enzymatic activity declined rapidly, with most lost within 1 h. A previous molecular dynamics simulation study suggested that increasing the temperature from 25 to 75 °C induces conformational changes in tyrosinase, particularly affecting the active site, leading to a loss of activity.^44^ Previous studies have demonstrated that white button mushroom tyrosinase remains stable at 30 °C for approximately 5 h but undergoes a sharp decline in activity as temperature increases beyond 45–55 °C.^34^ Similarly, *Lactarius piperatus* (L.) Pers. mushroom tyrosinase lost 20–60% of its activity within 4 h at temperatures ranging from 10 to 50 °C.^40^ *Macrolepiota gracilenta* tyrosinase lost 20% activity within 1 day at 4 °C and 60% activity at 30 °C.^23^

#### 3.2.3 Tyrosinase Kinetic Analysis

Figure 4 shows the Michaelis–Menten curve for mushroom stump tyrosinase activity as a function of increasing concentrations of l-DOPA (0.1–10 mM). The kinetic parameters, *K*_m_ and *V*_max_, were determined using the Lineweaver–Burk plot as 0.355 mM and 16.44 U/min, respectively. Mushroom stump tyrosinase exhibits a stronger affinity for l-DOPA (i.e., lower *K*_m_) than previously reported for mushroom cap/stem tyrosinase: *K*_m_ = 0.933 mM,^32^ and *K*_m_ = 0.87 mM,^43^ and also for a recombinant mushroom tyrosinase *K*_m_ = 26.1 mM.^20^ These variations in substrate affinity might be attributed to differences in the tyrosinase isoenzymes present. Notably, *Ab*PPO2 and *Ab*PPO5 exhibit *K*_m_ values of 0.32 ± 0.071 mM and 0.5 ± 0.026 mM,^7^ respectively, which are comparable to the *K*_m_ of mushroom tyrosinase in this study. In contrast, *Ab*PPO1, *Ab*PPO3, and *Ab*PPO5 have much higher *K*_m_ values, ranging from 3 to 6 mM, and *Ab*PPO6 demonstrates the lowest affinity for l-DOPA, with a *K*_m_ of 38 ± 3.6 mM.^7^ Since tyrosinase isomers exist in the crude enzyme extract, heterogeneity-aware models such as HetMM^45^ could be applied in future studies with expanded substrate ranges.

**Figure 4:**
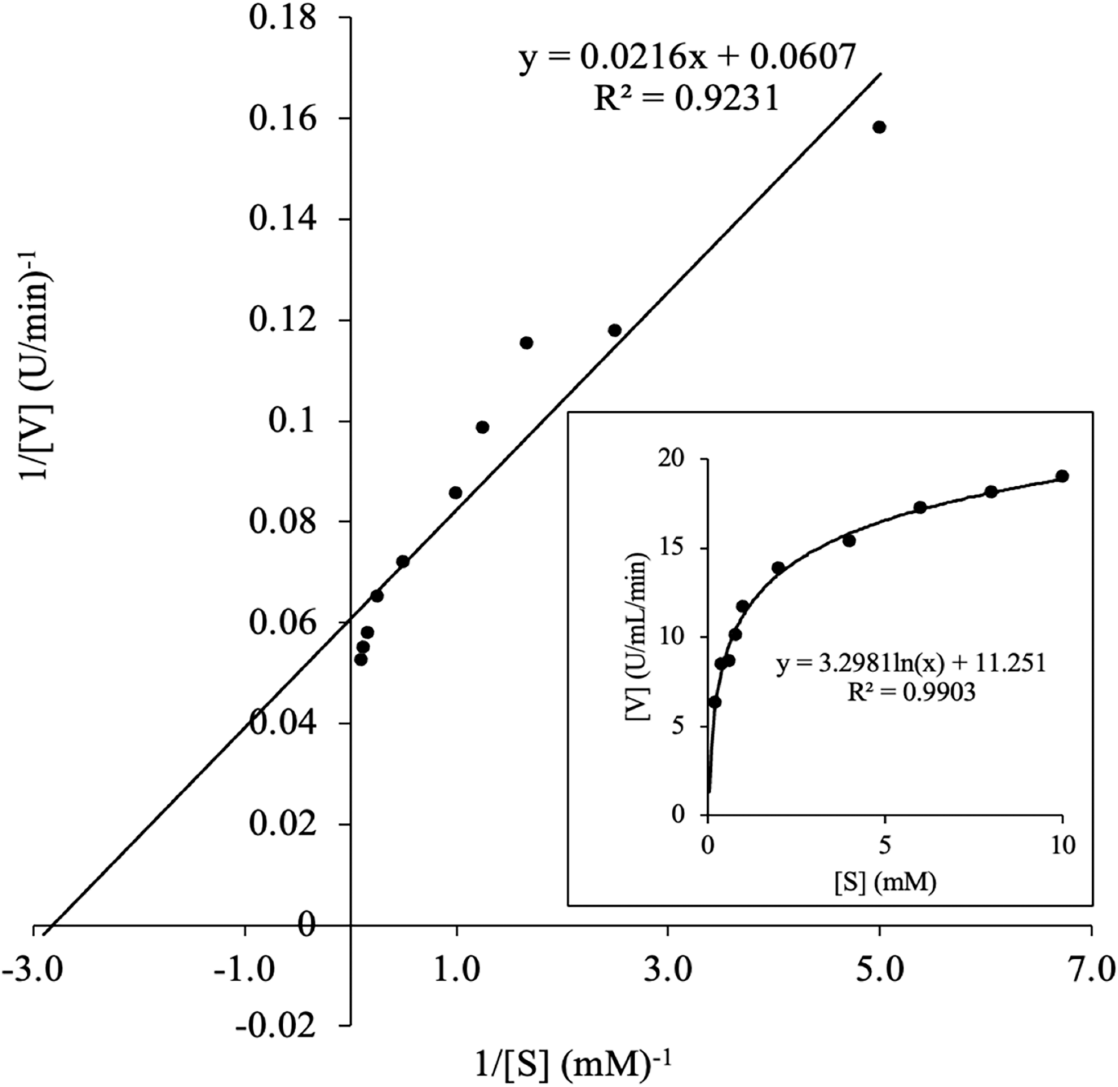
Lineweaver–Burke and Michaelis–Menten plots (inset) of crude tyrosinase with activity measured using l-DOPA as substrate.

#### 3.2.4 Effects of Chemical Reagents and Metal Ions on Tyrosinase Activity

Tyrosinase is a copper-dependent oxidase whose activity can be influenced by various metal ions and chemical reagents. Crude tyrosinase preparations also contain proteases and other components that may contribute to the observed effects of these reagents. Figure 5 shows that the addition of different chemical compounds has activation-, inhibition-, or concentration-dependent effects on tyrosinase activity. Among these, MnSO_4_ at 0.1–25 mM was found as a mild tyrosinase activator, with an increase in tyrosinase activity by 6 to 13% (Figure 5b). A previous study on actinomycete tyrosinases reported that addition of 0.1–5 mM Mn^2+^ either increased tyrosinase activity by 6% or had no effect for specific tyrosinase isomers.^46^

**Figure 5:**
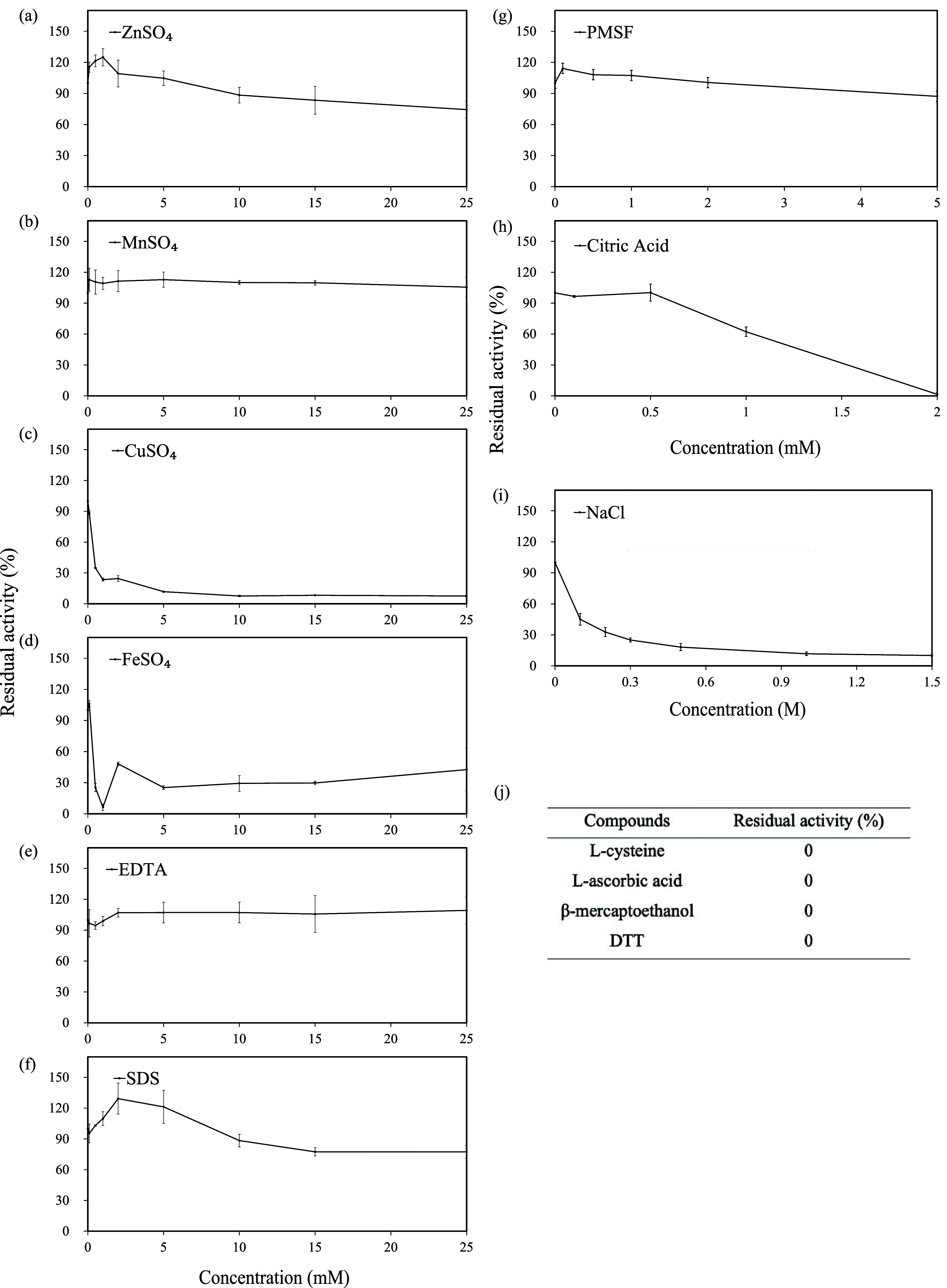
Effects of chemical reagents and metal ions on tyrosinase activity. (a) ZnSO_4_ (mM); (b) MnSO_4_ (mM); (c) CuSO_4_ (mM); (d) FeSO_4_ (mM); (e) EDTA (mM); (f) SDS (mM); (g) PMSF (mM); (h) citric acid (mM); (i) NaCl (M); (j) l-cysteine (0.1 mM), l-ascorbic acid (0.1 mM), β-mercaptoethanol (0.1 mM), and DTT (0.1 mM).

Seven compounds acted as tyrosinase inhibitors when added to crude tyrosinase: l-cysteine, l-ascorbic acid, β-mercaptoethanol (β-ME), DTT, CuSO_4_, NaCl, and citric acid (Figure 5c,h,i, and j). l-cysteine was reported to bind the tyrosinase Cu^2+^ and to trap the reaction product *o*-quinones, preventing pigment formation.^47^ l-ascorbic acid chelates copper and reduces *o*-quinones back to diphenols, therefore preventing dopachrome formation.^48^ β-ME and DTT are thiol-based reductants that disrupt the tertiary structure of tyrosinase by cleaving disulfide bonds and interacting with the copper center.^49^ CuSO_4_ was previously reported to strongly inhibit mushroom tyrosinase via binding that partially denatures tyrosinase, reducing its stability.^50^ As for NaCl, the Cl^–^ can interact with the enzyme–substrate complex, forming an enzyme–substrate-inhibitor complex that suppresses catalytic turnover.^51^ Citric acid was found to be a mild copper-chelator, inhibiting mushroom tyrosinase activity.^52^

Five compounds acted as concentration-dependent modifiers: ZnSO_4_, FeSO_4_, EDTA, SDS, and PMSF (Figure 5). ZnSO_4_ enhanced tyrosinase activity to 125% at 1 mM but inhibited it above 10 mM (Figure 5a). At low concentration, Zn^2+^ acts as a nonessential activator binding to the noncatalytic site to increase the *V*_max_ and stabilize its conformation.^53^ However, excess Zn^2+^ alters the conformation, reducing substrate accessibility and resulting in inhibitory effects.^54^ The effect of FeSO_4_ on tyrosinase activity fluctuated, suggesting that it interacted with unknown coexisting components that also play a role in regulating tyrosinase activity (Figure 5d). Fe^2+^ is generally considered an inhibitor to purified tyrosinase.^24^ EDTA is a strong chelating agent and has been reported to be a tyrosinase inhibitor due to chelating Cu^2+^. In this study, EDTA (0.1–25 mM) had a minor effect on tyrosinase (Figure 5e), which might be because the crude enzyme also contained other metal ions (Cu^2+^, Fe^2+^/Fe^3+^, Zn^2+^, Mn^2+^, etc.) and metalloproteinases,^55,56^ so EDTA might preferentially chelate these compounds. SDS enhanced tyrosinase activity at low concentrations (0.1–5 mM) but inhibited it at higher concentrations (Figure 5f). SDS was previously reported to activate latent tyrosinases by inducing limited conformational changes, but high concentrations or prolonged exposure times led to enzyme instability or even inactivation.^57,58^ PMSF (<0.5 mM) slightly enhanced tyrosinase activity but decreased when at 0.5–5 mM (Figure 5g). PMSF is a well-known inhibitor of serine proteases that are abundant in mushrooms and could degrade tyrosinase.^59^ In addition, a study reported that 1 mM PMSF reduced *Bacillus aryabhattai* tyrosinase activity to 93.6% and 5 mM PMSF reduced it to 79.9%.^24^ Overall, the chemical effects indicated that the coexisting components, possibly comprising metal ions, protease, and others, influenced the response of tyrosinase.

### 3.3 Tyrosinase Identification via Proteomics

Mushroom tyrosinases in latent form generally consist of two heavy chains at an MW of ∼43–45 kDa, and two light chains at an MW of ∼14–18 kDa, linked by covalent bonds.^6^ To confirm and identify the tyrosinases in crude enzymes, five protein bands on crude tyrosinase SDS-PAGE gel (heavy chains 1, 2, 3; light chains 1, 2) (Figure 1a) were subjected to proteomic analysis. The detected proteins of each band are listed in Tables S1 and S5, suggesting that numerous *Agaricus bisporus* mushroom proteins were present in crude tyrosinase. The tyrosinase-related proteins are given in Table 3. Overall, the crude tyrosinase contains AbPPO3, AbPPO4, and AbPPO5. The three heavy chains, especially heavy chain 2, matched the N-terminal region of the PPOs. The two light chains corresponded to the unique lectin-like protein identified as the light chain of tyrosinase; however, some studies recognized them as inevitable contamination proteins with tyrosinase.^36,60^

**Table 3:** Tyrosinases Identified via Proteomics

bands	accession	description	coverage [%]	# peptides	# Unique peptides	# PSMs	# AAs	MW [kDa]
Heavy Chain 1	C7FF04	polyphenol oxidase 3	45	20	19	62	576	66.2
	A0A8H7F118	tyrosinase	37	24	2	62	611	68.4
	K5XUX5	tyrosinase	37	24	2	60	610	68.3
	CAG9553209.1	AbPPO5_H39 *Agaricus bisporus* var. bisporus	39	22	21	45	576	66.1
Heavy Chain 2	CAG9553207.1	AbPPO4_H39 *Agaricus bisporus* var. bisporus	68	41	1	160	611	68.3
	A0A8H7F118	tyrosinase	65	40	2	156	611	68.4
	C7FF05	polyphenol oxidase 4	66	38	1	146	611	68.3
	C7FF04	polyphenol oxidase 3	62	28	27	94	576	66.2
	CAG9553209.1	AbPPO5_H39 *Agaricus bisporus* var. bisporus	51	25	7	79	576	66.1
	A0A8H7F178	tyrosinase	32	19	1	50	686	78.5
	CAG9553203.1	AbPPO2_H39 *Agaricus bisporus* var. bisporus	30	17	16	26	556	63.9
Heavy Chain 3	C7FF04	polyphenol oxidase 3	51	24	23	127	576	66.2
	A0A8H7F118	tyrosinase	32	19	2	43	611	68.4
	K5XUX5	tyrosinase	32	19	2	41	610	68.3
Light Chain 1	Q00022	*Agaricus bisporus* lectin	50	5	5	12	143	16.2
	G1K3P4	lectin-like fold protein	55	5	3	12	150	16.5
Light Chain 2	Q00022	*Agaricus bisporus* lectin	93	14	14	103	143	16.2
	C7FF04	polyphenol oxidase 3	40	17	16	44	576	66.2
	G1K3P4	lectin-like fold protein	62	7	3	14	150	16.5

It is worth noting that a protein band at an MW of ∼23 kDa was present as an impurity protein in both commercial and crude tyrosinases. Previous studies have found that commercial tyrosinase, despite additional purification steps, still contains impurities, such as other enzymes (e.g., proteases, laccase, glycosidase), proteins, carbohydrates, and phenols.^61–63^

### 3.4 Cross-Linking of Casein Using Tyrosinase

Tyrosinase enzymes have been used to cross-link proteins, thereby enhancing their functional properties.^64^ However, commercial tyrosinase is expensive for industrial uses. The crude extract in this study would be cheaper to prepare and, if functional, could be practical for food applications. To demonstrate this, the mushroom stump extract was used to catalyze the cross-linking of casein. This section is divided into three sections. Section 3.4.1: crude tyrosinase was added to casein and incubated at 30 °C for 120 min. However, protein profiles from SDS-PAGE analysis (Figure 6a) suggested the coexistence of proteases in crude tyrosinase that hydrolyzed casein. Section 3.4.2: tyrosinase, which had been partially purified via ammonium sulfate precipitation, was used to cross-link caseins (Figure 6b–e). Section 3.4.3: protease inhibitors (EDTA and PMSF) were added to the crude enzyme to inhibit the proteases and demonstrate the cross-linking reaction catalyzed by the tyrosinase present (Figure 7).

**Figure 6:**
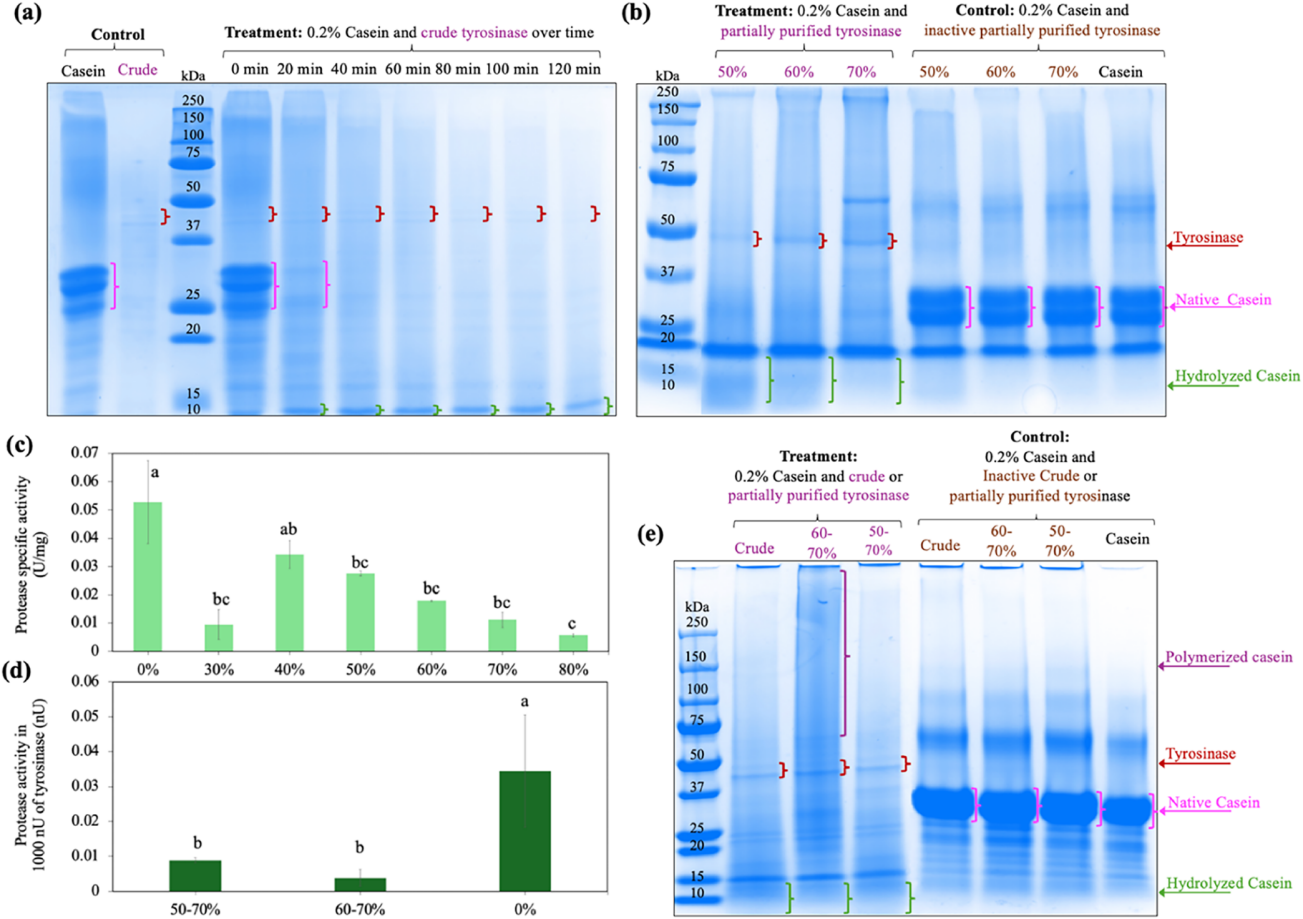
(a) SDS-PAGE profile of casein treated by crude tyrosinase every 20 min over 2 h, (b) SDS-PAGE profile of casein treated by partially purified tyrosinases, (c) specific activity of protease in the crude and partially purified tyrosinases, (d) ratio of protease in 1000 nU of aliquot tyrosinase, and (e) SDS-PAGE profile of casein treated by crude tyrosinase and partially purified tyrosinases (50–70%, 60–70% saturation fraction) for 1 h. Samples with different superscript letters are significantly different (*p* < 0.05).

**Figure 7:**
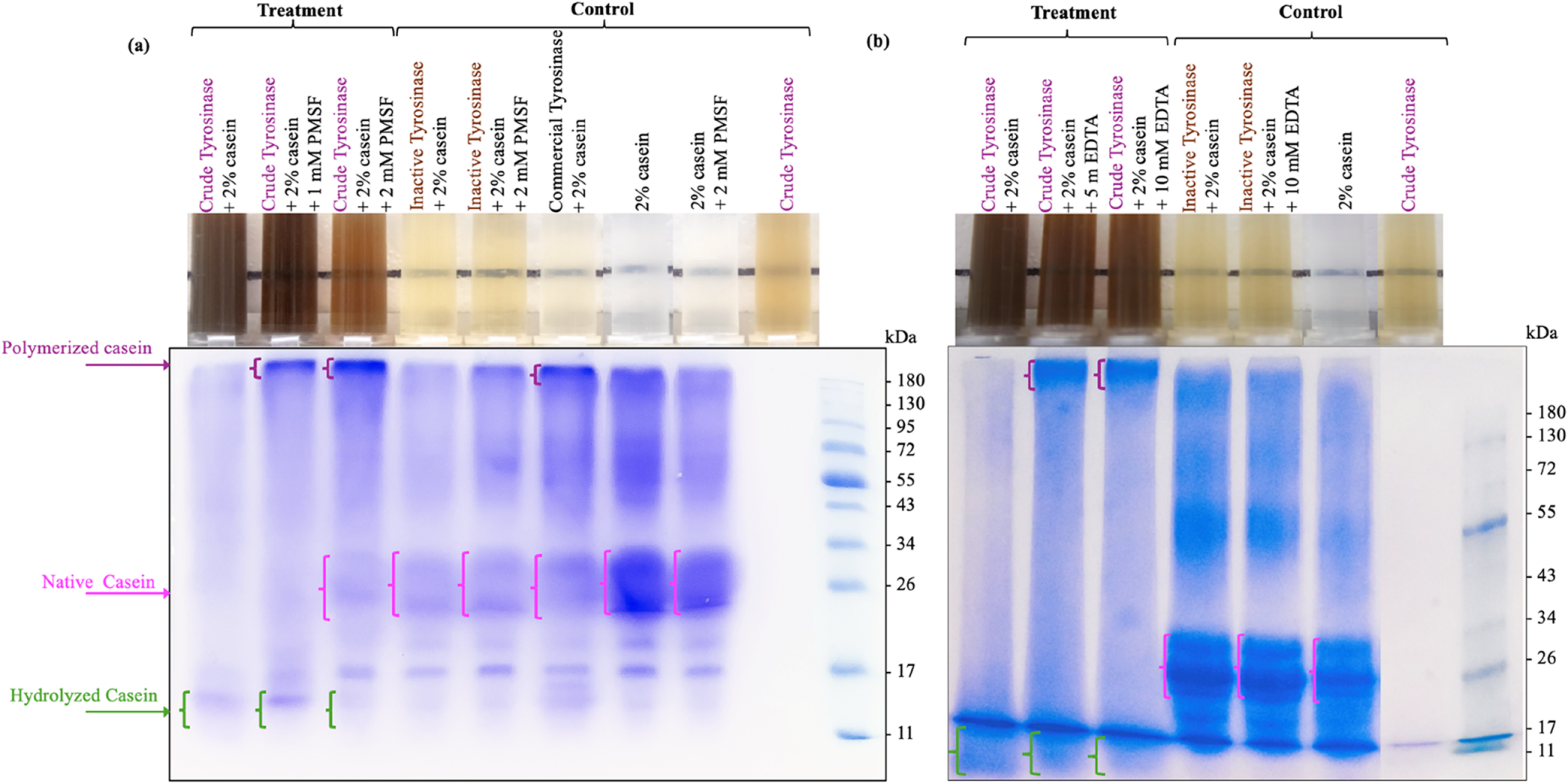
Reaction systems and the corresponding SDS-PAGE profiles regarding the effects of (a) PMSF and (b) EDTA on the inhibition of protease to preserve tyrosinase capacity in cross-linking casein.

#### 3.4.1 Effect of Coexisting Protease in Crude Tyrosinase

At the start of the reaction (0 min), 0.2% casein with 1000 nU of crude tyrosinase showed bands similar to those in the control (Figure 6a). However, after 20 min, the casein proteins were degraded into lower-molecular-weight bands (∼15–10 kDa), suggesting proteolysis. It seems that a protease in the crude enzyme solution rapidly hydrolyzed the casein, and any simultaneous cross-linking effect of the tyrosinase was not observed. In contrast, 2000 nU of the commercial tyrosinase (which presumably did not contain protease) produced high-molecular-weight casein complexes, confirming the enzyme’s ability to cross-link proteins (Figure 7a). The protease activity in the crude tyrosinase was measured as ∼0.05 U protease per 1 mg protein (Figure 6c). According to the literature, whole-genome sequencing of white button mushrooms identified 111 genes encoding proteases. These proteases play an important role in metabolism, allowing them to utilize protein as a carbon and nitrogen source in a nutrient-poor environment.^65,66^ Proteolysis by these endogenous proteases can cleave the N-terminal inhibitory prosegment of latent tyrosinase, converting it into its catalytically active form. In this study, the protease in the mushroom stump extract interfered with the assay, so there is no evidence that the tyrosinase present can cross-link casein proteins. Two strategies were employed to minimize the effect of protease relative to that of tyrosinase: partial purification via ammonium sulfate precipitation and protease inhibition.

#### 3.4.2 Use of Partially Purified Tyrosinase for Casein Cross-Linking

The presence of proteases in the crude mushroom stump extract made it impossible to demonstrate any cross-linking effect of the tyrosinase also present. Therefore, the ammonium sulfate fractions from Section 3.1.2. were used as they may at least partially separate tyrosinase from endogenous proteases. The protease and tyrosinase activities of the various ammonium sulfate fractions are shown in Figures 6c and 1b, respectively. The 30, 50, 60, 70, and 80% saturated fractions had significantly lower specific activity of protease compared with the crude extract. Among these, 50, 60, and 70% saturated fractions showed the highest tyrosinase-specific activity and the lowest protease-specific activity. Therefore, a combination of the 50, 60 and 70% saturated fractions (50–70%) was used for further casein cross-linking studies. SDS-PAGE analysis was used to demonstrate the capacity of these samples to hydrolyze casein (Figure 6b). The 50% saturated fraction caused more casein hydrolysis (i.e., greater reduction of the intensity of the initial casein band and greater development of lower-molecular-weight bands) than the 60 and 70% saturated fractions. Therefore, a combination of the 60 and 70% saturated fractions (60–70%) was used to cross-link casein in the experiment with the expectation that this combination might exhibit lower proteolytic activity.

The differences in the tyrosinase-specific activity of each fraction required the use of different sample volumes to obtain the same enzyme activity (1000 nU) in each. As a result, the amount of protease activity in each fraction was different in each sample (Figure 6d). The protease-to-tyrosinase activity ratio was highest in the crude extract, reflecting its high protease content (0.03 U). In contrast, the 50–70% and 60–70% saturated fractions required smaller volumes due to their higher tyrosinase activity and thus had lower protease activity (<0.01 U). The SDS-PAGE analysis (Figure 6e) showed that casein treated with crude tyrosinase, the 50–70% saturated fraction, and the 60–70% saturated fraction all developed bands with MW lower than 15 kDa (i.e., casein proteolysis), while other samples did not. However, the 60–70% saturated fraction also showed a new dark smear at higher MW (>75 kDa) from protein oligomers, suggesting that the caseins had been cross-linked by tyrosinase. The reason for the presence of a smear, instead of a band with a clear molecular weight, could be that the protease had partly hydrolyzed native casein into lower MW polypeptides (lower than 15 kDa), which are subsequently cross-linked by tyrosinase to form oligomers with a range of molecular weights. Alternatively, casein oligomers could be hydrolyzed by the present protease. While this experiment provides evidence that the tyrosinase in the crude mushroom stump extract can cross-link proteins, ammonium sulfate precipitation was insufficient to reduce protease activity for practical application. Therefore, a second series of casein cross-linking experiments was conducted using the crude extract in conjunction with protease inhibitors.

#### 3.4.3 Use of Protease Inhibitors to Preserve Tyrosinase-Mediated Casein Cross-Linking

According to the literature, in white button mushroom, there are 4 serine protease genes expressed at the highest up-regulated transcript, followed by metalloproteinase, aspartic acid, and cysteine protease genes.^67^ Proteases in commercial white button mushrooms were previously reported to be 100% inhibited by 1 mM PMSF and only 9.3% inhibited by 5 mM EDTA. PMSF is a well-established inhibitor of the serine proteases prevalent in mushrooms, which functions by irreversibly binding to the serine residue in the active site. However, it is not food-grade, and its use in food-related applications is limited. EDTA, in contrast, is not a potent inhibitor of serine proteases but is effective against metalloproteases by chelating the metal ions required for their catalytic activity.

Both EDTA and PMSF partially inhibited protease activity in the crude tyrosinase extract (Table S6). EDTA (10 mM) and PMSF (2 mM) inhibited approximately 30% and 40% of the protease activity, respectively. The degree of inhibition by PMSF was much less than that reported in the literature: Burton et al.^68^ showed that 1 mM PMSF was sufficient to completely inhibit white button mushroom proteases, while Heneghan et al.^69^ achieved a 95% reduction in activity with 100 mM PMSF. In contrast, the EDTA was more effective than reported previously: Burton et al.^68^ showed that 5 mM EDTA only inhibited white button mushroom proteases by 9.3%. This difference may be due to the presence of multiple types of proteases in the crude extract, with metalloproteases present being inhibited by the EDTA and the serine proteases by PMSF. The weaker effect of EDTA compared to PMSF may reflect the lower expression of metalloprotease genes compared to serine protease genes in *Agaricus bisporus*.^67^

The impact of protease inhibition on tyrosinase activity and casein cross-linking is illustrated in Figure 7. High-molecular-weight protein bands near the top of the gel were observed in samples treated with crude tyrosinase in the presence of PMSF or EDTA, indicating successful casein polymerization. In contrast, casein treated with crude tyrosinase without any added inhibitor showed complete hydrolysis of casein and no high-molecular-weight bands. Gels from the PMSF (2 mM) samples had the clear native casein bands and more intense high-molecular-weight bands (Figure 7a, Lane 3), indicating more effective protease suppression and enhanced tyrosinase-driven cross-linking compared to EDTA. This is consistent with the results showing that PMSF is more effective than EDTA in inhibiting protease in crude mushroom stump extract (Table S6) and with the literature reports that serine proteases are predominant in mushrooms. Protease inhibitors were effective in suppressing proteolytic degradation, allowing for visualization of tyrosinase-mediated casein polymerization. Although PMSF was the most effective in suppressing protease activity, its nonfood-grade status and associated safety and cost concerns limit its applicability in food systems. Conversely, EDTA, being both food-grade and moderately effective, represents a more viable candidate for enabling protein cross-linking using mushroom stump extracts in food-related applications.

#### 3.4.4 Molecular Mechanism of Casein Cross-Linking Using Crude Tyrosinase

The casein cross-linking observed in this study can occur via one of two pathways, depending on whether a phenolic mediator is present or absent (Figure S1).^70^ In the absence of a phenolic mediator, cross-linking can occur via a 1,4-addition reaction or radical coupling, involving direct tyrosine oxidation. Alternatively, in the presence of a low-molecular-weight phenolic mediator, the 1,4-addition reaction of the *o*-quinone mediators to thiol/amine amino acid residues becomes the preferred mechanism, and the mediator enhances the efficiency of covalent bond formation between protein molecules. In this study, the casein samples containing the crude extract quickly turned brown, indicating that the tyrosinase was oxidizing endogenous phenolic compounds extracted from the mushrooms. In contrast, samples containing the commercial tyrosinase did not turn brown, suggesting that purification had removed all of the phenolic compounds (Figure 7). The phenolic compounds in the crude extract could act as internal mediators for protein cross-linking. In addition, the action of protease enzymes in the crude extract on casein may expose hidden tyrosine and cysteine residues, which act as a substrate for tyrosinase to catalyze cross-linking reactions. We speculate that these two factors in the crude extract, endogenous phenolics and limited proteolysis, enhanced the tyrosinase-mediated protein polymerization (Figure 7). If this is the case, then the crude tyrosinase, with natural polyphenols and some residual protease activity, could be a promising enzyme preparation for food applications involving protein cross-linking.

In conclusion, this study successfully demonstrated the recovery of tyrosinase from white button mushroom waste. The crude tyrosinase exhibited an optimal pH of 7.5 and maintained stability across an alkaline pH range (7.5–10.0). The enzyme exhibited optimal activity at 45 °C and remained stable between 4 and 30 °C. Tyrosinase activity was significantly inhibited by 5 mM concentrations of β-ME, DTT, l-ascorbic acid, and l-cysteine. In comparison, it was enhanced by the presence of 5 mM ZnSO_4_, MnSO_4_, SDS, and citric acid. Ammonium sulfate precipitation at 50–70% saturation yielded the highest tyrosinase recovery (48%) from the crude extract, establishing a helpful initial step for further purification. Furthermore, crude tyrosinase, when treated to reduce protease activity either by ammonium sulfate precipitation or with protease inhibitors (PMSF and EDTA), retained the ability to catalyze the cross-linking of casein. These findings provide valuable insights into the potential dual benefit of reducing agriculture waste and repurposing it into a low-cost source of biocatalyst for protein modification. However, further investigations are necessary to explore cost-effective purification methods. Additionally, the mechanisms of enzyme action, substrate specificity, and scale-up production are crucial for their application in food processing.

## Supplementary Material



## Notes

Mechanism of protein cross-linking by tyrosinase catalysis via (a) tyrosine amino acids and (b) phenolic compound; pH and thermal stability of crude tyrosinase; proteomic analysis of tyrosinase heavy chain 1; proteomic analysis of tyrosinase heavy chain 2; proteomic analysis of tyrosinase heavy chain 3; proteomic analysis of tyrosinase light chain 1; proteomic analysis of tyrosinase light chain 2; and the effect of inhibitors on protease activity (PDF)

## Abbreviations Used

l-DOPA: l-phenylalanine
PMSF: phenylmethanesulfonyl fluoride
EDTA: ethylenedinitrilotetraacetic acid
β-ME: β-mercaptoethanol
DTT: DL-dithiothreitol
SDS-PAGE: sodium dodecyl sulfate–polyacrylamide gel electrophoresis
BCA: bicinchoninic acid
BSA: bovine serum albumin
(PPO): polyphenol oxidase
