# Supplementary material for: Tyrosinase Recovered from White Button Mushroom Waste: Extraction, Characterization, and Application in Casein Cross-Linking
Source: J Agric Food Chem. 2026 Mar 10;74(10):8550–62. doi: 10.1021/acs.jafc.5c16655 (PMC13003501; doi:10.1021/acs.jafc.5c16655)
Supplement: Supplementary file 1 [file jf5c16655_si_001.pdf]

Supporting Information

Tyrosinase Recovered from White Button Mushroom Waste: Extraction, Characterization and Application in Casein Crosslinking

Trang Thuy Tran, Zhe Xu, John Coupland & Yi Zhang *

Department of Food Science, The Pennsylvania State University, University Park, PA, 16802, USA

* Corresponding author:

Yi Zhang, PhD. Email: yjz5549@psu.edu

Other authors:

Trang Thuy Tran. Email: ttt5333@psu.edu

Zhe Xu. Email: zxx5155@psu.edu

John Coupland, PhD. Email: jnc3@psu.edu

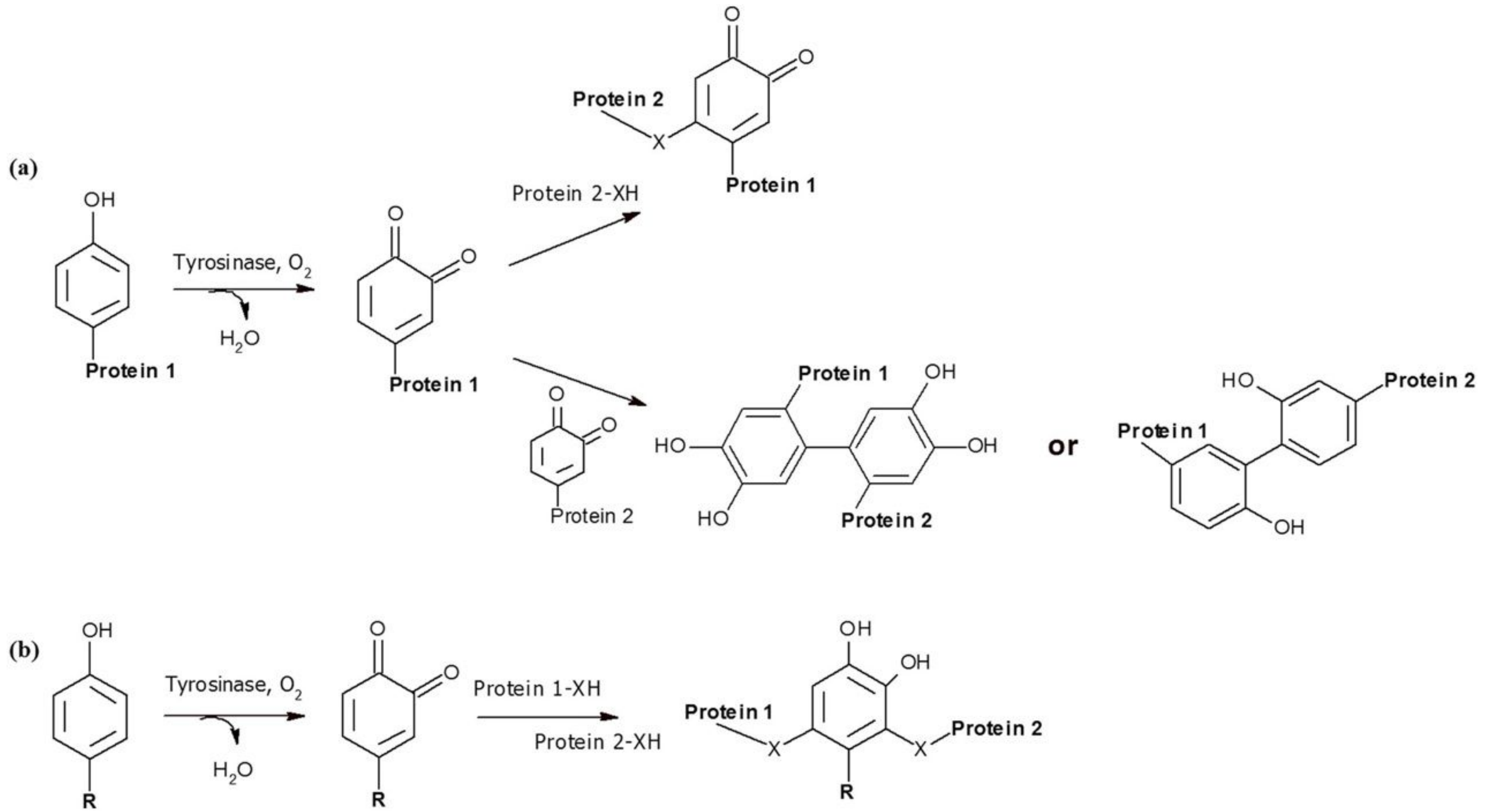

Fig. S1. Mechanism of protein crosslinking by tyrosinase catalysis via (**a**) Tyrosine amino acids, and (**b**) Phenolic compound. Adapted with permission from Isaschar-Ovdat, S., & Fishman, A. (2018). Crosslinking of food proteins mediated by oxidative enzymes–a review. Trends in Food Science & Technology, 72, 134-143. Copyright 2018. Elsevier.

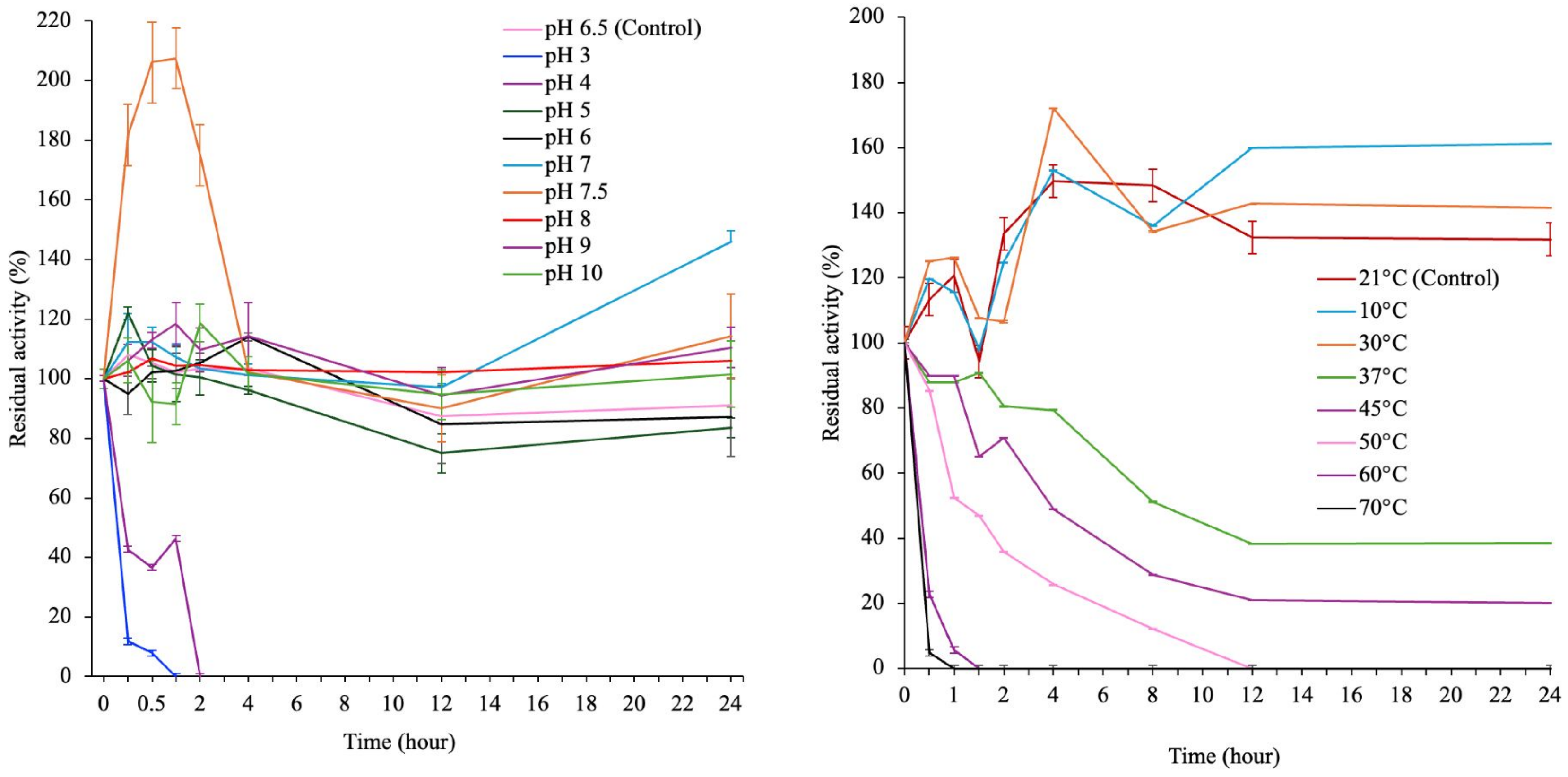

Fig. S2. pH and thermal stability of crude tyrosinase.

Table S1. Proteomic analysis of tyrosinase heavy chain 1.

Accession	Description	Coverage [%]	# Peptides	# Unique Peptides	# PSMs	# AAs	MW [kDa]
A0A8H7F8V9	Phosphoglycerate mutase-like	45	17	17	115	444	50
A0A8H7KIB3	Glutathione reductase	67	29	29	92	543	59.2
K5Y7K9	phosphopyruvate hydratase	68	22	1	79	444	47.4
A0A8H7FA35	phosphopyruvate hydratase	68	22	1	76	444	47.4
A0A8H7KKN1	NADH:flavin oxidoreductase	63	25	2	77	406	44.6
K5Y813	NADH:flavin oxidoreductase	59	24	1	75	402	44.2
A0A8H7BYD9	Elongation factor 1-gamma	43	18	18	83	416	46.8
C7FF04	Polyphenol oxidase 3	45	20	19	62	576	66.2
K5X9C9	Multifunctional fusion protein	50	24	2	54	546	59.7
P78568	Delta-1-pyrroline-5-carboxylate	48	23	1	53	546	59.7
A0A8H7F118	tyrosinase	37	24	2	62	611	68.4
K5XUX5	tyrosinase	37	24	2	60	610	68.3
P04264	Keratin, type II cytoskeletal 1	45	26	20	47	644	66
P54387	NADP-specific glutamate	50	18	18	41	457	49.5
A0A8H7F5H2	Aminopeptidase	44	33	33	46	895	99.5
A0A8H7C107	Extracellular metalloproteinase	22	16	16	41	783	86.6
A0A8H7F7T5	2-methylcitrate dehydratase	53	23	23	44	481	53.3
CAG9553209.1	AbPPO5_H39 Agaricus bisporus	39	22	21	45	576	66.1
K5XJL4	Cysteine proteinase 1,	35	16	2	39	509	56.7
K5X2U9	Glycoside hydrolase family 5	36	16	16	33	474	54.8
A0A8H7KJG6	Cysteine proteinase 1,	35	15	1	37	497	55.5
P13645	Keratin, type I cytoskeletal 10	39	21	18	37	584	58.8
K5W1X6	Phosphoenolpyruvate	47	21	21	33	564	63.6
K5WXN3	Large ribosomal subunit protein	40	17	17	37	388	43.8
K5VVN4	Aminotransferase class I/classII	47	19	19	33	476	52.2
K5X1X7	FAD-binding PCMH-type	31	11	3	31	503	54.7
K5X3K8	Aldehyde dehydrogenase domain-	48	21	16	35	502	54.7
P35527	Keratin, type I cytoskeletal 9	37	18	17	31	623	62

K5X606	Peptidase M20 domain-containing	35	14	13	32	438	48
P35908	Keratin, type II cytoskeletal 2	42	23	16	30	639	65.4
A0A8H7EWU9	Adenosylhomocysteinase	42	16	16	29	430	47.1
A0A8H7KIV3	Transcriptional coregulator SSA1	36	22	21	30	654	71.1
A0A8H7KHU4	CAZyme family GT4	35	20	20	29	737	82
K5X1N5	phosphoglucomutase (alpha-D-	43	20	20	30	576	62.4
K5XJ37	Pyruvate kinase	43	18	18	28	546	59.8
K5X516	homogentisate 1,2-dioxygenase	43	15	15	27	478	53.3
K5X8L3	Dihydrolipoyl dehydrogenase	36	15	15	25	508	54
K5XYI5	FAD-binding PCMH-type	42	14	14	22	468	50.8
A0A8H7BXR6	Heat shock 70 kDa protein C	32	19	19	25	675	73.4
A0A8H7F356	Protein disulfide-isomerase	30	12	12	22	520	57.6
K5Y601	methylmalonate-semialdehyde	41	16	16	19	546	58.2
P13647	Keratin, type II cytoskeletal 5	34	21	11	25	590	62.3
A0A8H7F1Y5	Formamidase	48	13	13	21	423	45.4
A0A8H7BZ28	Chaperonin GroL	33	17	17	19	604	63.1
K5X548	Phosphotransferase	40	15	15	19	497	54.9
K5Y1D3	Aminotransferase class V domain-	30	10	10	18	427	46.9
A0A8H7EWU7	Serine hydroxymethyltransferase	32	14	14	23	480	52.5
A0A8H7KHF3	Phosphotransferase	38	16	16	21	507	54.1
A0A8H7KGN9	Rieske domain-containing protein	36	17	17	18	573	61.8
K5WU13	Large ribosomal subunit protein	36	12	12	16	371	41.2
K5X583	Peptidase M20 dimerisation	33	11	11	12	475	52.2
A0A8H7FA58	TauD/TfdA-like domain-	35	11	11	12	395	44.3

Table S2. Proteomic analysis of tyrosinase heavy chain 2.

Accession	Description	Coverage [%]	# Peptides	# Unique Peptides	# PSMs	# AAs	MW [kDa]
A0A8H7FA35	phosphopyruvate hydratase	72	28	1	150	444	47.4
A0A8H7KKN1	NADH:flavin oxidoreductase	69	30	2	155	406	44.6
K5Y7K9	phosphopyruvate hydratase	71	29	2	149	444	47.4
K5Y813	NADH:flavin oxidoreductase	66	29	1	149	402	44.2
CAG9553207.1	AbPPO4_H39 Agaricus bisporus	68	41	1	160	611	68.3
A0A8H7F118	tyrosinase	65	40	2	156	611	68.4
P54387	NADP-specific glutamate	63	25	25	132	457	49.5
C7FF05	Polyphenol oxidase 4	66	38	1	146	611	68.3
C7FF04	Polyphenol oxidase 3	62	28	27	94	576	66.2
K5Y1D3	Aminotransferase class V domain-	60	21	21	78	427	46.9
CAG9553209.1	AbPPO5_H39 Agaricus bisporus	51	25	7	79	576	66.1
A0A8H7F503	non-chaperonin molecular chaperone	45	30	29	83	614	67.3
A0A8H7F8V9	Phosphoglycerate mutase-like	52	20	20	78	444	50
A0A8H7KIV3	Transcriptional coregulator SSA1	39	25	24	68	654	71.1
K5WXN3	Large ribosomal subunit protein uL3	53	24	24	73	388	43.8
A0A8H7EWU9	Adenosylhomocysteinase	53	22	13	63	430	47.1
K5XT06	Peptidase M24 domain-containing	65	26	26	59	396	43
A0A8H7KG82	Galactose mutarotase-like protein	74	24	24	52	418	45.8
A0A8H7C107	Extracellular metalloproteinase	30	18	18	46	783	86.6
A0A8H7F178	tyrosinase	32	19	1	50	686	78.5
A0A8H7BYD9	Elongation factor 1-gamma	43	18	18	50	416	46.8
K5WSK2	Peptidase M20 domain-containing	49	18	4	35	437	47.8
K5XJL4	Cysteine proteinase 1, mitochondrial	48	18	3	37	509	56.7
P78568	Delta-1-pyrroline-5-carboxylate	46	21	2	34	546	59.7
A0A8H7KIJ7	Dipeptidyl peptidase 3	43	24	24	34	699	77.9
A0A8H7CAU6	alanine--glyoxylate transaminase	39	16	16	35	386	41.6
K5X9C9	Multifunctional fusion protein	42	20	1	33	546	59.7
K5X606	Peptidase M20 domain-containing	42	18	17	34	438	48

A0A8H7KJG6	Cysteine proteinase 1, mitochondrial	44	16	1	34	497	55.5
A0A8H7C631	Isocitrate dehydrogenase [NADP]	43	19	19	34	454	51.3
A0A8H7EXI1	Peptidase M20 domain-containing	43	15	1	30	437	47.8
A0A8H7BZY5	Aldehyde dehydrogenase domain-	41	16	12	30	502	54.7
K5XJ37	Pyruvate kinase	55	22	22	31	546	59.8
K5XXS7	Peptidase A1 domain-containing	44	13	4	29	413	44.9
P02533	Keratin, type I cytoskeletal 14	50	19	8	28	472	51.5
Q9P8B4	Glucuronyl hydrolase (Fragment)	46	13	13	27	400	43.9
K5WAZ8	Glycoside hydrolase family 5	48	20	20	30	423	48.3
P13645	Keratin, type I cytoskeletal 10	35	18	15	25	584	58.8
K5W8D0	Glutathione reductase	42	17	17	23	471	51.3
K5XYI5	FAD-binding PCMH-type domain-	38	13	13	23	468	50.8
A0A8H7F5H2	Aminopeptidase	33	21	21	23	895	99.5
CAG9553203.1	AbPPO2_H39 Agaricus bisporus	30	17	16	26	556	63.9
P04264	Keratin, type II cytoskeletal 1	31	19	16	24	644	66
A0A8H7KHM7	PABS domain-containing protein	31	18	12	24	759	83.6
A0A8H7F8W0	Heat shock protein 70	32	18	18	22	798	87.9
A0A8H7KHP7	Prolyl endopeptidase	30	17	17	23	727	82.6
K5WU13	Large ribosomal subunit protein uL4	47	17	17	24	371	41.2
A0A8H7BZ28	Chaperonin GroL	36	19	19	21	604	63.1
K5X516	homogentisate 1,2-dioxygenase	36	12	12	21	478	53.3
K5W1X6	Phosphoenolpyruvate carboxykinase	35	15	15	19	564	63.6
K5VK04	Alpha-galactosidase	33	9	9	18	410	44.6
A0A8H7F0X2	Survival factor 1	42	12	12	18	426	46.6
K5XXV9	NAD(P)-binding domain-containing	45	10	10	15	343	37.2
P08779	Keratin, type I cytoskeletal 16	31	13	2	18	473	51.2
A0A8H7EWU7	Serine hydroxymethyltransferase	33	14	14	19	480	52.5
K5XNK9	Oxysterol-binding protein	35	10	10	15	392	43.5
A0A8H7KGU5	Citrate synthase	33	13	13	17	462	50.6
K5WLE0	Isomerase YbhE	30	6	6	12	361	38.3
A0A8H7C8X3	phosphoglucomutase (alpha-D-	36	15	15	15	565	61.3

K5VVN4	Aminotransferase class I/classII	37	14	14	14	476	52.2
A0A8H7KJM9	Formate dehydrogenase	33	10	10	13	368	40.1
A0A8H7F1Y5	Formamidase	32	8	8	9	423	45.4
K5Y799	Proteasome subunit beta	48	7	7	8	208	22.9

Table S3. Proteomic analysis of tyrosinase heavy chain 3.

Accession	Description	Coverage [%]	# Peptides	# Unique	# PSMs	# AAs	MW [kDa]
A0A8H7KIV3	Transcriptional coregulator SSA1	51	31	30	129	654	71.1
C7FF04	Polyphenol oxidase 3	51	24	23	127	576	66.2
A0A8H7EWU9	Adenosylhomocysteinase	56	23	13	100	430	47.1
P04264	Keratin, type II cytoskeletal 1	55	33	28	87	644	66
K5WU13	Large ribosomal subunit protein uL4	56	20	19	80	371	41.2
P35527	Keratin, type I cytoskeletal 9	56	27	26	54	623	62
A0A8H7CAU6	alanine--glyoxylate transaminase	48	17	17	60	386	41.6
K5XXS7	Peptidase A1 domain-containing	46	14	4	56	413	44.9
A0A8H7KJT0	Iron-sulfur cluster biogenesis	31	17	17	49	692	75
K5X9C9	Multifunctional fusion protein	43	22	2	49	546	59.7
P78568	Delta-1-pyrroline-5-carboxylate	42	21	1	47	546	59.7
K5XT06	Peptidase M24 domain-containing	55	20	20	51	396	43
Q9P8B4	Glucuronyl hydrolase (Fragment)	40	12	12	42	400	43.9
P35908	Keratin, type II cytoskeletal 2	46	25	19	40	639	65.4
Q711G1	Glucose-6-phosphate isomerase	45	21	21	37	551	61.4
K5X4V7	Gfo/Idh/MocA-like oxidoreductase	49	12	2	39	377	39.4
K5W1X6	Phosphoenolpyruvate carboxykinase	52	23	23	37	564	63.6
K5WYA1	Adenosylhomocysteinase	32	16	6	47	430	47.3
P54387	NADP-specific glutamate	47	18	18	38	457	49.5
A0A8H7C4Z3	Gfo/Idh/MocA-like oxidoreductase	50	11	1	33	377	39.4
K5Y813	NADH:flavin oxidoreductase	52	18	1	35	402	44.2
A0A8H7KKN1	NADH:flavin oxidoreductase	52	18	1	34	406	44.6
A0A8H7F118	tyrosinase	32	19	2	43	611	68.4
P13645	Keratin, type I cytoskeletal 10	44	22	16	34	584	58.8
K5XUX5	tyrosinase	32	19	2	41	610	68.3
K5XJ37	Pyruvate kinase	57	24	24	35	546	59.8
K5X9E4	3-isopropylmalate dehydrogenase	56	16	2	32	381	40.3
A0A8H7F2L0	3-isopropylmalate dehydrogenase	56	15	1	29	372	39.4

K5XXV9	NAD(P)-binding domain-containing	54	14	1	28	343	37.2
A0A8H7KGW6	NAD(P)-binding domain-containing	54	14	1	28	343	37.2
K5X043	Amidohydrolase-related domain-	39	13	13	30	410	46.1
A0A8H7F9L2	6-phosphogluconolactonase	37	12	12	26	419	45.7
A0A8H7BYD9	Elongation factor 1-gamma	32	13	13	27	416	46.8
A0A8H7F8W0	Heat shock protein 70	34	20	20	27	798	87.9
K5XNK9	Oxysterol-binding protein	32	10	10	24	392	43.5
K5VK04	Alpha-galactosidase	35	10	10	22	410	44.6
K5X516	homogentisate 1,2-dioxygenase	37	13	13	24	478	53.3
K5X3K8	Aldehyde dehydrogenase domain-	30	13	10	22	502	54.7
K5W8D0	Glutathione reductase	44	15	15	22	471	51.3
A0A8H7KGU5	Citrate synthase	30	12	12	20	462	50.6
P02533	Keratin, type I cytoskeletal 14	34	15	4	19	472	51.5
K5Y441	Aspartate aminotransferase	36	12	12	20	410	45.2
A0A8H7F5C1	TauD/TfdA-like domain-containing	39	16	15	20	391	44.1
K5XYI5	FAD-binding PCMH-type domain-	31	12	10	17	468	50.8
K5X2Q8	GST C-terminal domain-containing	37	11	11	17	321	36.6
A0A8H7C3A5	ornithine carbamoyltransferase	33	10	2	17	378	41.7
A0A8H7F0X2	Survival factor 1	35	10	10	14	426	46.6
K5XL39	NADP-dependent oxidoreductase	45	13	13	13	363	40.7
K5XH02	NADP-dependent oxidoreductase	43	10	10	12	288	31.8
K5WAZ8	Glycoside hydrolase family 5	31	10	10	11	423	48.3

Table S4. Proteomic analysis of tyrosinase light chain 1.

Accession	Description	Coverage [%]	# Peptides	# Unique Peptides	# PSMs	# AAs	MW [kDa]
B3GW73	Fruit-body specific gene D (Fragment)	64	4	4	58	99	11.1
P04264	Keratin, type II cytoskeletal 1	48	27	19	46	644	66
P13645	Keratin, type I cytoskeletal 10	45	23	18	31	584	58.8
P35908	Keratin, type II cytoskeletal 2 epidermal	36	19	12	25	639	65.4
K5X226	Histone H4 (Fragment)	57	7	7	25	94	10.5
A0A8H7F7Z0	YCII-related domain-containing protein	70	7	7	20	113	12.6
K5XUX5	tyrosinase	28	17	1	25	610	68.3
K5WUK5	Yeast cell wall synthesis Kre9/Knh1-like	61	6	6	21	127	13.8
K5XSY8	ACB domain-containing protein	68	7	7	24	101	11.5
K5WZB8	Peptidase A1 domain-containing protein	70	5	5	24	101	11
K5Y7K9	phosphopyruvate hydratase	36	11	11	22	444	47.4
O93868	NADP-dependent mannitol	32	9	9	20	262	28
A0A8H7EZR4	Ribosomal protein	64	6	6	18	115	12.1
K5W801	Nucleoside diphosphate kinase	64	7	7	18	151	16.7
K5XL39	NADP-dependent oxidoreductase	33	8	8	14	363	40.7
K5WX98	Translationally-controlled tumor protein	46	6	6	18	168	19
A0A8H7F539	L-dopachrome isomerase	43	6	6	16	120	13.6
Q00022	Agaricus bisporus lectin	50	5	5	12	143	16.2
K5XV53	Cythochrome C	57	7	7	13	108	11.8
K5X434	Ribosomal protein L38e	46	5	5	14	82	9.5
G1K3P4	Lectin-like fold protein	55	5	3	12	150	16.5
K5VXA7	DUF427 domain-containing protein	76	5	1	13	100	11.5
A0A8H7EZG5	Cytidine deaminase	48	5	5	13	159	17
A0A8H7C2R6	Peptidyl-prolyl cis-trans isomerase	52	8	8	16	162	17.5
A0A8H7FC10	60S ribosomal protein L8	35	11	11	16	268	29.5
A0A8H7F354	Ubiquitin-like domain-containing	32	5	5	15	147	16.6
A0A8H7BYD9	Elongation factor 1-gamma	32	10	10	13	416	46.8
K5W785	Ketoreductase domain-containing	43	9	9	15	272	29.1

K5WIS3	Small nuclear ribonucleoprotein Sm D1	37	4	4	12	122	13.6
A0A8H7EZP1	Dynein light chain	47	4	4	10	109	12.1
K5Y042	40S ribosomal protein S8	38	7	7	12	216	24.3
K5WPY6	Histone H2B	50	8	2	14	147	15.5
K5X2Q8	GST C-terminal domain-containing	32	9	9	14	321	36.6
A0A8H7KIX8	GTP-binding protein ypt1	46	9	9	12	204	22.6
K5XA58	60S ribosomal protein L36	31	4	4	11	100	11.4
A0A8H7C724	DUF427 domain-containing protein	66	5	1	11	100	11.5
A0A8H7KIS5	Histone H2B	42	7	1	13	143	15.2
A0A8H7C7S2	Proteasome subunit beta	30	6	6	11	256	28.5
K5WW43	GST N-terminal domain-containing	41	8	8	12	246	28
A0A8H7C7U7	Lipoprotein	37	3	3	11	105	11.6
A0A8H7EZW4	60S ribosomal protein L43	35	5	5	9	126	14
K5XCZ7	NADP-dependent oxidoreductase	30	9	6	10	325	36.8
K5XQC8	peptidylprolyl isomerase	43	4	4	9	108	11.9
K5XCN6	40S ribosomal protein S18	42	9	9	12	156	18
A0A384E160	Protein AB21	54	9	9	10	208	22.5
A0A8H7EVV9	Uncharacterized protein	31	2	1	7	121	13
A0A8H7C6C6	NADP-dependent oxidoreductase	38	9	9	9	324	36.8
K5XK56	Small nuclear ribonucleoprotein Sm D2	33	4	4	10	111	12.9
K5XFY0	Large ribosomal subunit protein	36	6	6	9	186	20.9
A0A8H7KL91	Uncharacterized protein	31	6	6	9	216	24.3
K5W6G4	Thioredoxin domain-containing protein	46	5	5	7	156	17.5
A0A8H7F9B1	RRM domain-containing protein	41	3	3	5	146	14.4
A0A8H7FAB9	40S ribosomal protein S9	35	8	8	10	191	21.9
A0A8H7KJK9	LSM complex subunit LSm2	68	6	6	7	96	11.1
K5X5Q8	Spliceosomal protein DIB1	33	4	4	6	142	16.7
K5X728	Plectin/eS10 N-terminal domain-	30	6	6	8	147	16.8
A0A8H7F8S1	Uncharacterized protein	43	4	4	7	106	11.9

Table S5. Proteomic analysis of tyrosinase light chain 2.

Accession	Description	Coverage [%]	# Peptides	# Unique Peptides	# PSMs	# AAs	MW [kDa]
Q00022	Agaricus bisporus lectin	93	14	14	10	143	16.2
K5W801	Nucleoside diphosphate kinase	80	11	11	86	151	16.7
K5XD35	Peptidase S8/S53 domain-containing	40	12	12	55	492	52.1
C7FF04	Polyphenol oxidase 3	40	17	16	44	576	66.2
K5XLF5	KOW domain-containing protein	59	11	11	40	136	15.3
K5VKT6	Ribosomal protein L14b/L23e	64	8	8	27	137	14.5
P78568	Delta-1-pyrroline-5-carboxylate	40	16	1	26	546	59.7
A0A8H7C	Peptidyl-prolyl cis-trans isomerase	54	8	8	27	162	17.5
K5XRX2	Calmodulin	62	9	9	23	149	16.7
K5X9C9	Multifunctional fusion protein	38	16	1	26	546	59.7
A0A8H7F3	Ubiquitin-like domain-containing	32	4	4	23	147	16.6
P04264	Keratin, type II cytoskeletal 1	30	16	14	21	644	66
B3GW73	Fruit-body specific gene D	64	4	4	16	99	11.1
A0A8H7F8	Uncharacterized protein	51	7	7	24	106	11.9
A0A8H7K	NADH:flavin oxidoreductase	35	12	12	17	406	44.6
A0A8H7K	Uncharacterized protein	42	6	6	20	184	20
A0A8H7KI	Transcriptional coregulator SSA1	32	18	17	22	654	71.1
A0A384E1	Protein AB21	70	12	5	20	208	22.5
K5WXA3	Large ribosomal subunit protein uL18	36	11	11	19	307	35
K5VUV6	60S ribosomal protein L31	60	8	8	20	126	14.3
A0A8H7E	Ribosomal protein	64	7	7	16	115	12.1
A0A8H7E	Adenosylhomocysteinase	33	11	11	16	430	47.1
A0A8H7F9	RRM domain-containing protein	42	3	3	12	146	14.4
O93868	NADP-dependent mannitol	30	8	8	16	262	28
K5WXJ5	Large ribosomal subunit protein uL23	49	10	10	19	158	17.6
A0A8H7E	Cytidine deaminase	67	7	7	15	159	17
A0A8H7K	Proteasome subunit alpha type	47	12	12	18	263	29

G1K3P4	Lectin-like fold protein	62	7	3	14	150	16.5
K5XA58	60S ribosomal protein L36	54	6	6	15	100	11.4
K5XXQ3	Yeast cell wall synthesis Kre9/Knh1-	42	3	3	9	124	13.2
P46792	Small ribosomal subunit protein uS8	48	5	5	14	130	14.7
A0A8H7C	Profilin	61	6	6	15	135	14.4
A0A8H7B	40S ribosomal protein S14	41	7	7	14	150	16
A0A8H7F	Ribosomal protein L22e	55	5	5	12	122	13.8
A0A8H7B	Elongation factor 1-gamma	33	12	12	14	416	46.8
A0A8H7K	Glycan binding protein Y3-like	32	4	4	12	134	14
A0A8H7KI	Histone H2B	42	6	1	13	143	15.2
K5WPY6	Histone H2B	50	7	2	13	147	15.5
A0A8H7F9	Uncharacterized protein	44	8	1	12	195	20.9
K5WJX7	NADP-dependent oxidoreductase	31	8	4	11	347	39
A0A8H7E	HIT domain-containing protein	43	5	4	10	136	15.3
A0A8H7E	Uncharacterized protein	31	2	1	9	121	13
K5WU68	Ricin B lectin domain-containing	34	5	1	11	150	16.5
A0A8H7E	Dynein light chain	51	5	5	9	109	12.1
K5WZ40	Uncharacterized protein	31	2	1	7	122	13.3
K5XFG9	Sm protein B	34	8	8	11	178	18.9
K5WZB8	Peptidase A1 domain-containing	39	4	4	11	101	11
K5WY21	Proteasome subunit alpha type	30	7	7	8	277	30.2
A0A8H7E	Histone H2A	34	4	2	10	138	14.4
A0A8H7F	40S ribosomal protein S9	32	9	9	11	191	21.9
Q9HGX4	Histone H2A	34	4	2	9	139	14.7
A0A8H7F	5-hydroxyisourate hydrolase	31	4	4	7	132	14.7
K5XFY0	Large ribosomal subunit protein	36	6	6	7	186	20.9
A0A8H7F5	Ricin B lectin domain-containing	38	5	1	7	146	16.5
A0A8H7K	Proteasome subunit alpha type	35	7	7	7	242	26.9
K5X373	Ricin B lectin domain-containing	39	5	1	6	146	16.4
K5X226	Histone H4 (Fragment)	55	5	5	6	94	10.5
K5WWE0	Uncharacterized protein	34	4	4	4	170	18

K5XCN6	40S ribosomal protein S18	32	6	6	6	156	18
A0A8H7C	Peptide-methionine (R)-S-oxide	52	6	6	6	132	14.3
A0A8H7C	Thioredoxin domain-containing	30	4	4	4	182	19.1
K5WZP3	Small nuclear ribonucleoprotein Sm	30	3	3	3	128	14

Table S6. Effect of inhibitors on protease activity.

Inhibitors	Residual activity (%)
10 mM EDTA	70
2 mM PMSF	60
